# *Cyclopia* extracts act as selective estrogen receptor subtype downregulators in estrogen receptor positive breast cancer cell lines: Comparison to standard of care breast cancer endocrine therapies and a selective estrogen receptor agonist and antagonist

**DOI:** 10.3389/fphar.2023.1122031

**Published:** 2023-03-13

**Authors:** Folasade R. Olayoku, Nicolette J. D. Verhoog, Ann Louw

**Affiliations:** Department of Biochemistry, Stellenbosch University, Stellenbosch, South Africa

**Keywords:** *Cyclopia*, honeybush, tea extract, selective estrogen receptor subtype downregulator, ERα, ERβ

## Abstract

Breast cancer is the most diagnosed type of cancer amongst women in economically developing countries and globally. Most breast cancers express estrogen receptor alpha (ERα) and are categorized as positive (ER^+^) breast cancer. Endocrine therapies such as, selective estrogen receptor modulators (SERMs), aromatase inhibitors (AIs), and selective estrogen receptor downregulators (SERDs) are used to treat ER^+^ breast cancer. However, despite their effectiveness, severe side-effects and resistance are associated with these endocrine therapies. Thus, it would be highly beneficial to develop breast cancer drugs that are as effective as current therapies, but less toxic with fewer side effects, and less likely to induce resistance. Extracts of *Cyclopia* species, an indigenous South African fynbos plant, have been shown to possess phenolic compounds that exhibit phytoestrogenic and chemopreventive activities against breast cancer development and progression. In the current study, three well characterized *Cyclopia* extracts, SM6Met, cup of tea (CoT) and P104, were examined for their abilities to modulate the levels of the estrogen receptor subtypes, estrogen receptor alpha and estrogen receptor beta (ERβ), which have been recognized as crucial to breast cancer prognosis and treatment. We showed that the *Cyclopia subternata* Vogel (*C. subternata* Vogel) extracts, SM6Met and cup of tea, but not the *C. genistoides* extract, P104, reduced estrogen receptor alpha protein levels while elevating estrogen receptor beta protein levels, thereby reducing the ERα:ERβ ratio in a similar manner as standard of care breast cancer endocrine therapies such as fulvestrant (selective estrogen receptor downregulator) and 4-hydroxytamoxifen (elective estrogen receptor modulator). Estrogen receptor alpha expression enhances the proliferation of breast cancer cells while estrogen receptor beta inhibits the proliferative activities of estrogen receptor alpha. We also showed that in terms of the molecular mechanisms involved all the *Cyclopia* extracts regulated estrogen receptor alpha and estrogen receptor beta protein levels through both transcriptional and translational, and proteasomal degradation mechanisms. Therefore, from our findings, we proffer that the *C. subternata* Vogel extracts, SM6Met and cup of tea, but not the *C. genistoides* extract, P104, selectively modulate estrogen receptor subtypes levels in a manner that generally supports inhibition of breast cancer proliferation, thereby demonstrating attributes that could be explored as potential therapeutic agents for breast cancer.

## 1 Introduction

Breast cancer (BC) is the most diagnosed type of cancer as well as the major source of cancer-associated deaths amongst women globally (DeSantis et al., 2015; DeSantis et al., 2019). The burden of disease is rapidly growing in economically developing countries with over half (52%) of new BC cases and 62% of mortalities occurring within this region (DeSantis et al., 2015). Roughly 70% of BCs express ERα and are categorized as ER^+^ BC (Gonzalez et al., 2019). The most common endocrine treatments for ER^+^ BC thus target either ER signaling, *via* SERMs and SERDs, or the production of estrogen, *via* AIs (Rozeboom et al., 2019; Costa et al., 2020).

The effects of estrogen in breast cancer are mediated by two ER subtypes, ERα and ERβ. ERα regulates the genes involved in cell proliferation, differentiation, and migration in mammary tissue *via* endocrine and paracrine mechanisms (Hartman et al., 2009; Leung et al., 2012; Huang et al., 2015; Saha et al., 2019). Interestingly, the role of ERβ in BC is still elusive since ERβ functions differently depending on the availability of ERα (Girgert et al., 2019). ERβ has generally been shown to facilitate apoptosis as well as to counter the proliferative activity of ERα in healthy mammary tissue (Huang et al., 2015). Furthermore, the level of ERβ and its co-expression with ERα has been suggested to modulate the cell’s response to estrogen in BC cell lines and may also modulate the response of ER^+^ BC to endocrine therapy (Song et al., 2019; Mal et al., 2020; Datta et al., 2022). Thus, ERβ should be considered as a potential target for the treatment of BC (Gustafsson and Warner, 2000; Nilsson et al., 2011; Hirao-Suzuki, 2021).

The current study is motivated by the limitations associated with most adjuvant endocrine therapies developed to combat BC and the need to develop novel drugs that while effective, are less toxic, demonstrate fewer side effects, and are less likely to induce resistance (Clarke et al., 2015; Ramani et al., 2017; Sayed et al., 2019; Szostakowska et al., 2019; Franzoi et al., 2021). ERα has been identified as a viable drug target in resistant BC and thus the development of SERD therapies that specifically target the elimination of ERα is of considerable interest all the more so as fulvestrant, the only SERD currently approved by the FDA, suffers from poor oral bioavailability and has to be administered intramuscularly (Nathan et al., 2017; Shagufta et al., 2020; Downton et al., 2022; Farkas et al., 2022). Moreover, novel natural products or extracts provide possibilities for the discovery of new cancer therapies, especially for BC, as a substantial number of anticancer drugs currently used in the clinic are of natural origin (Zink and Traidl-Hoffmann, 2015; Wangkheirakpam, 2018; Yang et al., 2021).

Traditional medicine involves the long historical use of natural products and their derivatives as herbal medicines or therapy for diseases based on ancient cultural theories and practices (Gurib-Fakim, 2006; Chintamunnee and Mahomoodally, 2012), with plants being the main source of medication (van Wyk and Prinsloo, 2018). The 2019 World Health Organization (WHO) global report on traditional and complementary medicine (T&CM) shows an increase in public interest and acceptance and indicates that the practice is mostly accepted in Africa (WHO Report, 2019), especially amongst the population in rural areas (Dalglish et al., 2019). Although T&CM has gained global recognition (Ekor, 2014; Tahvilian et al., 2014; Lopes et al., 2017; Wang K. et al., 2021) its use is still limited by a lack of quality evidence-based research (Pelkonen et al., 2014; Pal, 2021; Veziari et al., 2021).

Often, traditional medicinal products are consumed as diet or as food supplements (Mbendana et al., 2019) and in South Africa, some dietary plants such *Aspalathus linearis* (rooibos tea), *Cyclopia* species (honeybush tea) and *Athrixia phylicoides* (bush tea) are considered medicinal herbal teas (Joubert et al., 2008). Extracts of *A. linearis* and *A. phylicoides* demonstrate assorted medicinal attributes, as do extracts from *Cyclopia* species, the major focus of the current study (Joubert et al., 2008; Louw et al., 2013; Joubert et al., 2019) Specifically, *Cyclopia* species, such as *C. subternata* Vogel, *C. genistoides C. sessiliflora*, *C. intermedia*, *C*. *longifolia*, and *C. maculata,* demonstrate anti-diabetic (Chellan et al., 2014; Schulze et al., 2016), anti-obesity (Pheiffer et al., 2013; Jack et al., 2018), and immune-stimulatory activities (Murakami et al., 2018) and osteoclast formation inhibition (Visagie et al., 2015); in addition to their useful application in nutraceutical, and cosmetic products (Joubert et al., 2019). Particularly of relevance to the current study, the *C. subternata* Vogel extract, SM6Met, was shown in several studies to possess phytoestrogenic activity, to display ERα antagonism and ERβ agonism, to antagonize estrogen-induced proliferation in ER^+^ BC cells (Mfenyana et al., 2008; Louw et al., 2013; Visser et al., 2013; Mortimer et al., 2015; van Dyk, 2018) and to ameliorate BC in rats (Visser et al., 2016; Oyenihi et al., 2018). Like SM6Met, the cup of tea (CoT) extract from *C. subternata* Vogel and the *C. genistoides* extract, P104, also exhibit phytoestrogenic properties and antagonize estrogen-induced proliferation in ER^+^ BC cells (Verhoog et al., 2007b; Visser et al., 2013; Roza et al., 2017).

The current study focusses on the assessment of the potential SERD activities of the *Cyclopia* extracts, SM6Met, CoT and P104, *via* the ER subtypes, ERα and ERβ, in BC cell lines. We hypothesize that the *Cyclopia* extracts may function as selective ER subtype regulators, thus, selectively affecting the levels of ER subtypes.

## 2 Materials and methods

### 2.1 Cell culture

The human BC cell line, MCF7-BUS (Soto et al., 1995) was kindly donated by Ana Soto, department of Anatomy and Cell biology, Tufts University School of Medicine, and the T47D cell line (Keydar et al., 1979) was a generous donation from Iqbal Parker, Medical biochemistry division, University of Cape Town. The two cell lines were maintained at 37°C with 5% CO2 and 90%–95% humidity in cell maintenance medium, which consisted of Dulbecco’s Modified Eagle’s Medium (DMEM) containing 4.5 g/mL glucose (Sigma-Aldrich, South Africa) supplemented with 5% (v/v) heat-inactivated fetal calf serum (HI-FCS) (The Scientific Group, South Africa), 1.5 g/L sodium-bicarbonate, 0.11 g/L sodium-pyruvate and 1% penicillin-streptomycin (100 IU/mL penicillin and 100 μg/mL streptomycin, Sigma-Aldrich) for MCF7 cells. For T47D cells the maintenance medium was the same except for 10% FCS used. The cell lines were routinely tested for *mycoplasma* by Hoechst staining and found to be negative. Experiments were carried out on cell lines with passage numbers between 6–30.

### 2.2 Test panel

The estrogenic compounds and *Cyclopia* extracts that make up the test panel include the endogenous hormone control, 17β-estradiol (E_2_), and the standard of care endocrine therapies (SOCs), (2)-4-hydroxytamoxifen (4-OHT) as a SERM control (Jordan, 2003) and fulvestrant (Ful) as a SERD control (Nathan et al., 2017), which were obtained from Sigma-Aldrich. The ER subtype selective ligands, methylpiperidinopyrazole (MPP), a ERα antagonist (Zhou et al., 2009), and liquiritigenin (Liq), a ERβ agonist (Mersereau et al., 2008), were purchased from Tocris Bioscience. The *Cyclopia* extracts, SM6Met, cup of tea (CoT), and P104 were obtained from cultivated and commercially harvested plant material and were previously prepared and characterized (Table 1). Retention samples of all the extracts have been preserved.

**TABLE 1:** Major polyphenols present in previously prepared *Cyclopia* extracts as determined by HPLC.

Polyphenolic compounds present in *Cyclopia* extracts	(g/100 g dry extract)^a^		
	SM6Met^b^	CoT^b^	P104^c^
Mangiferin	1.899	1.000	3.606
Isomangiferin	0.645	0.420	5.094
Luteolin	0.040	0.018	0.096
Scolymoside (7-*O*-rutinosylluteolin)	1.289	0.876	nd^f^
Vicenin-2 (6,8-di-β-D-glucopyranosylapigenin)	0.089	0.065	nd
Eriocitrin (7-*O*-rutinosylerodictyol)	0.846	0.600	nd
Hesperidin (7-*O*-rutinosylhesperetin)	2.049	0.935	nd
3′,5′-di-β-D-Glucopyranosylphloretin	1.278	0.939	nd
3′,5′-di-β-D-Glucopyranosyl-3-hydroxyphloretin^d^	0.700	0.582	nd
3-β-D-Glucopyranosyliriflophenone	0.669	0.590	nd
3-β-D-Glucopyranosyl-4-*O*-β-D-glucopyranosyliriflophenone^e^	0.958	0.896	nd
Protocatechuic acid	0.113	0.082	nd

^a^ g/100 g dry extracts denotes the quantity (g) of the polyphenolic compound present in 100 g of *Cyclopia* extract.

^b^ Prepared from *Cyclopia subternata* Vogel harvesting, M6, harvested on 30 March 2004 from a commercial plantation at Kanetberg farm near Barrydale, South Africa. Four batches (B1-B4) of SM6Met were prepared in 2012 by Mortimer et al. (2015) and Visser et al. (2013). Batches 1-4 were mixed in equal weights to prepare 3 mixes. Mix 2 was used in the current study. The CoT extract (batch 1) was also prepared in 2012 by Mortimer et al. (2015) and Visser et al. (2013) from the M6 harvesting.

^c^ Prepared from *Cyclopia genistoides* harvested on 15 March 2001 from a commercial plantation at Koksrivier, Pearly beach, South Africa. The P104 extract used for the current study was prepared by Verhoog et al. (2007b) and Verhoog et al. (2007a).

^d^ Structure unambiguously elucidated by Human et al. (2021).

^e^ Structure unambiguously elucidated by Beelders et al. (2014).

^f^ Nd—polyphenolic compounds not detected due to absence or trace amounts.

Stock solutions of the test panel were prepared in DMSO (Sigma-Aldrich) and stored at −20°C until use (see Supplementary Table S1). Stock solutions were diluted 1000X in treatment media, (phenol red-free DMEM (low glucose), 1.5 g/L sodium bicarbonate and 3.5 g/L glucose) to yield a final concentration of 0.1% DMSO.

### 2.3 Western blot

MCF7 and T47D cells were plated at 1.0 × 10^5^ cells/well into 12 well plates and steroid starved in steroid starving media (phenol red-free DMEM (low glucose) supplemented with 1.5 g/L sodium bicarbonate, 3.5 g/L glucose, 1% penicillin-streptomycin and 5% heat-inactivated doubly dextran-coated charcoal-stripped FCS (2xDCCFCS) for MCF7 cells and 10% 2xDCCFCS for T47D cells for 24 h. MCF7 and T47D cells were then washed in pre-warmed phosphate-buffered saline (PBS) and treated a with increasing concentrations of the test panel for 24 h in the treatment medium.

Following treatment, the treated MCF7 and T47D cells were washed in 1 mL ice-cold PBS and lysed in 100 μL Radioimmunoprecipitation assay (RIPA) buffer [50 mM Tris-HCl, 150 mM NaCl, 1% (v/v) NP40, 1% (w/v) sodium deoxycholate and 0.1% (w/v) SDS]. The lysates were transferred into Eppendorf tubes and 5x SDS reducing buffer [100 mM Tris-HCl pH 6.8, 50% (v/v) SDS, 20% (v/v) glycerol, 2% (v/v) β-mercaptoethanol and 0.1% (w/v) bromophenol blue] was added to enhance cell lysis. Thereafter the cell lysates were boiled at 95°C for 20 min.

To separate the proteins, the sodium dodecyl sulphate polyacrylamide gel electrophoresis (SDS-PAGE) technique was employed, where 10 μL of the lysate was loaded onto a 15 well 10% acrylamide gel containing 0.9% (v/v), 2,2,2- trichloroethanol (TCE), both procured from Sigma-Aldrich. A protein molecular weight marker (color pre-stained protein standard, broad range from Inqaba Biotec) was loaded alongside the lysates to verify the sizes of ERα and ERβ protein. The gel was set to run at 75 V for 15 min and at 150 V for 60 min.

The separated proteins on the acrylamide gel were imaged under UV light and the image acquired using the BioRad molecular imager, Gel DocTM XR+ with Image LabTM software. The acquired image of the total protein content was utilized for normalization (as detailed below). The proteins were then transferred to a Hybond-ECL nitrocellulose membrane (Separation Scientific) under 0.18 A electric current for 2 h. To ensure a successful protein transfer, the nitrocellulose membrane and the gels were imaged and acquired under UV light with the BioRad molecular imager, Gel DocTM XR+ with Image LabTM software after the transfer (see Supplementary Figure S1). The gels were then discarded, and the membranes were blocked in 10% milk powder at room temperature for 90 min on a Stovall Belly dancer shaker. The membranes were then washed consecutively for 15 min and 5 min with 1x Tris-buffered saline tween (TBST) [50 mM Tris base, 150 mM NaCl and 0.1% (v/v) Tween 20 dissolved in deionized water], followed by a 5 min wash with 1x Tris-buffered saline (TBS) (50 mM Tris base and 150 mM NaCl dissolved in deionized water). Thereafter, the membranes were probed with the primary antibody {anti-ERα [sc-8oo2 (F-10), Santa Cruz Biotechnology and anti- ERβ (MA524807/PPZ0506], Thermo Fisher Scientific} at 4°C overnight on a Stovall Belly dancer shaker. The membranes were then washed consecutively for 15 and 5 min in TBST, and in TBS for 5 min. After washing, the membranes were incubated with the secondary antibody (Rabbit anti-mouse IgG H&L (HRP) ab97046 from Abcam) for 90 min at room temperature on a Stovall Belly dancer shaker. Once more, the membranes were washed as described previously. The membranes were then incubated with BioRad ECL Western blotting reagent for 5 min and imaged using the iBrightTM Imaging System from Invitrogen (see Supplementary Figure S1).

To quantify the intensity of the ERα and ERβ protein bands, MyImage Analysis software was used. Equal protein loading was ensured by normalization to the total protein content of each lane. The total protein content was obtainable by adding a sedative agent, TCE (Chopra et al., 2019), to the SDS-PAGE gels. The TCE attaches hydroxyethanone to the indole ring of tryptophan residues that results in the fluorescence of protein bands under UV light, which was quantified using transilluminator molecular imager (BioRad molecular imager, Gel DocTM XR+ with Image LabTM software). The total protein of the test panel-treated lysates was set relative to that of the vehicle. The normalization factor (NF) of the vehicle was set at 1, in which case a NF < 1 or NF > 1 indicates that the total protein content of the test panel-treated lysate is higher or lower than that of the vehicle, respectively. The intensity of the ERα and ERβ protein bands was then multiplied by the NFs to obtain the normalized intensity of the band. Normalized ER expression was plotted as a percentage (average ± SD) relative to the vehicle (DMSO) sample, which was set to 100%. Dose-response curves were generated by fitting experimental values to the three-parameter logistic curve fitting equation in GraphPad Prism with the maximal response constrained to 100% to obtain the efficacy (maximal response) and potency (IC_50_). Supplementary Figure S1 contains an example of the full SDS-PAGE gel following protein separation, the full nitrocellulose membrane after protein transfer, the full SDS-PAGE gel after protein transfer and the full nitrocellulose membrane following immunoblotting (the Western blot).

#### 2.3.1 Proteasomal and translational inhibition

To study the effects of proteasomal and translational inhibition on the modulation of ERα and ERβ protein levels by the test panel the proteasomal inhibitor, MG132 (Fan et al., 2004) and the translational inhibitor, cycloheximide (CHX) (Schneider-Poetsch et al., 2010) were obtained from Sigma-Aldrich. Stock solutions for both inhibitors were prepared in DMSO and both were used at a final concentration of 1 nM.

### 2.4 Statistical analysis

Statistical analysis was carried out using GraphPad Prism software version 5. Details of the individual statistical analysis used, including post-tests are described in the figure legends. Statistical difference is expressed as either a different letter or using symbols (*, ^#^ and ^$^), as specified in the figure legends. Non-significant results are denoted by “ns.” For all Figures Average ± SD is of three independent biological experiments analyzed as such.

## 3 Results

### 3.1 Subtype selective modulation of ER subtype protein levels by *Cyclopia* extracts in MCF7 and T47D cell lines

**FIGURE 1:**
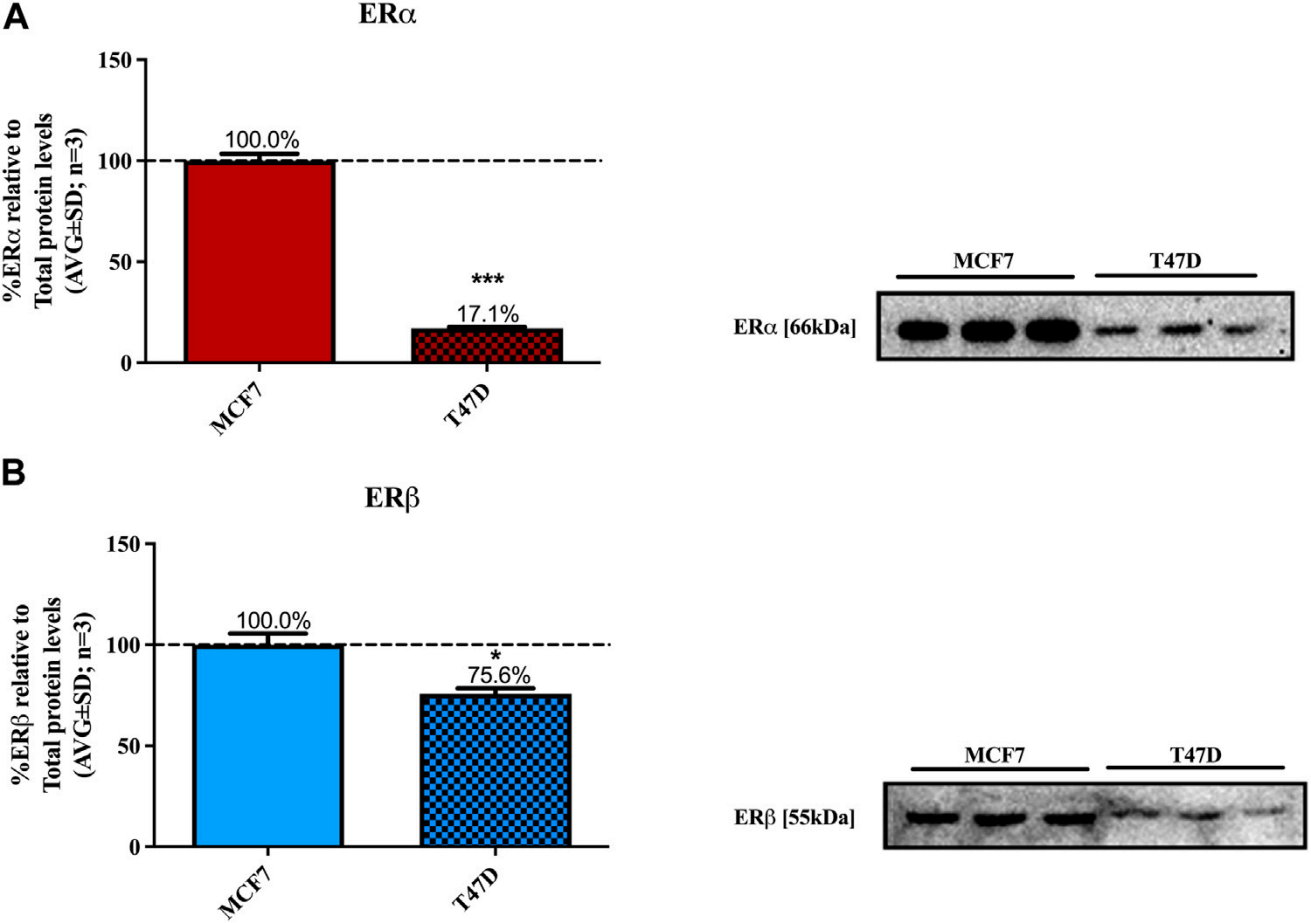
Basal levels of ERα and ERβ protein in MCF7 and T47D cells. MCF7 and T47D cells were steroid starved for 24 h. The media was then changed to high glucose-DMEM only and the cells incubated for another 24 h, after which ERα and ERβ basal protein levels were determined using Western blot. The western blots shown are representatives of three independent experiments for **(A)** ERα and **(B)** ERβ. For quantification, the intensity of the ERα and ERβ bands were determined with MyImage Analysis software, after which the obtained values were normalized to total protein content and expressed as a percentage (AVG ± SD) of MCF7 ERα and ERβ protein levels, which was set at 100% (black dotted line). WB is not an absolutely quantitative technique (i.e., the absolute concentrations of proteins cannot be ascertained) only the relative amounts of a protein may be compared between cell lines. Furthermore, as two different antibodies were used for ERα and ERβ we cannot directly compare the absolute levels for the ER subtypes. Thus, we chose to normalize the ER subtype levels to that in MCF7 cells. Statistical analysis was done using unpaired *t*-test (*p < 0.05, ***p < 0.0001) to evaluate the statistical difference between the basal levels of ERα and ERβ protein in MCF7 and T47D cells.

MCF7 and T47D cell lines are considered acceptable models for ERα^+^ luminal A carcinomas (Lacroix & Leclercq, 2004). They require estrogen for proliferation and although both cell lines express ERα and ERβ, MCF7 has a high ERα/ERβ ratio and T47D has a low ERα/ERβ ratio (Nadal-Serrano et al., 2012) as confirmed in Figure 1.

**FIGURE 2:**
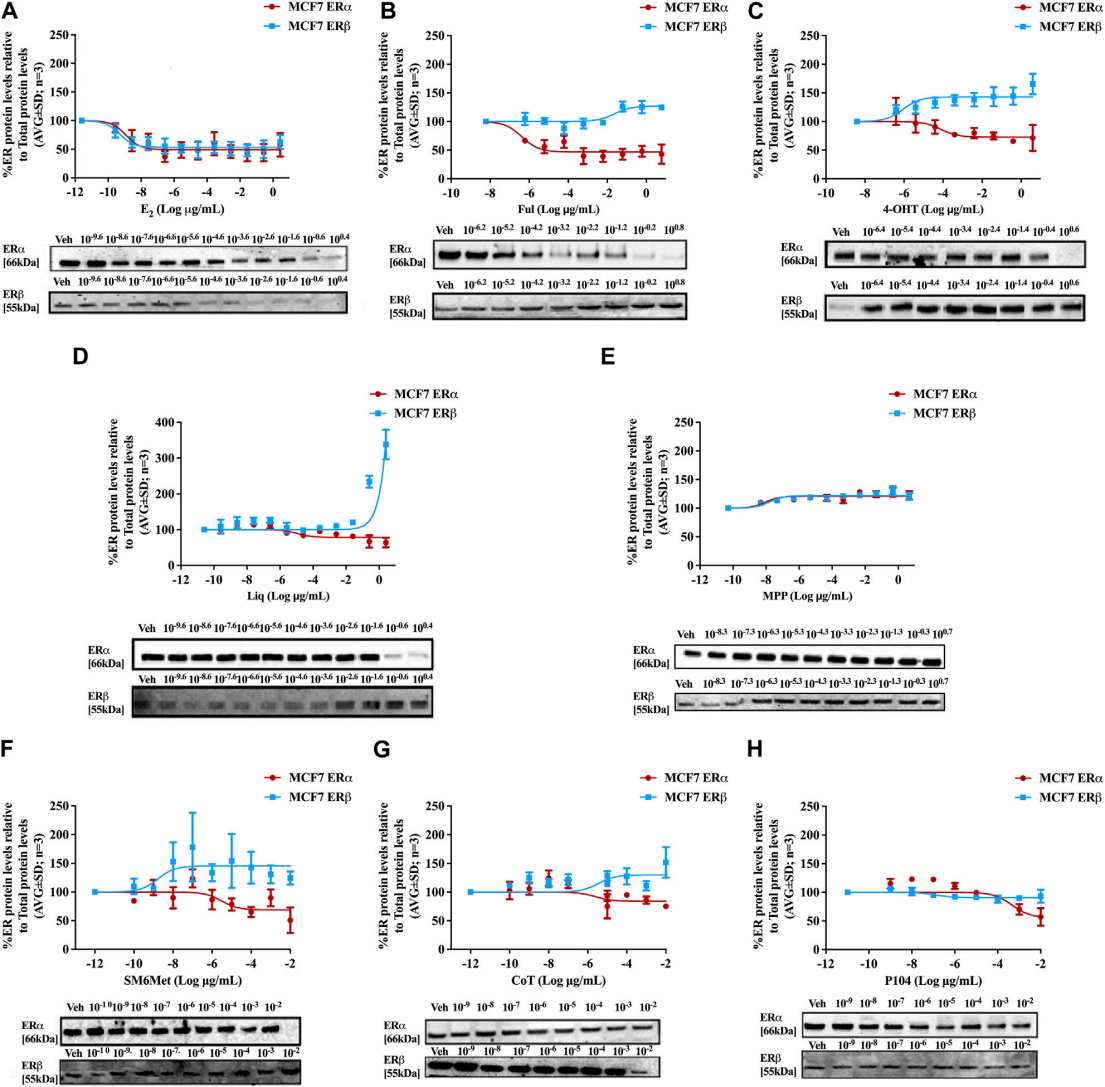
Effect of test panel on ERα and ERβ protein levels in MCF7 cells. MCF7 cells were steroid starved for 24 h and then treated with either vehicle (DMSO) or increasing concentrations of **(A)** E2, the SOCs, **(B)** fulvestrant (Ful) or **(C)** hydroxytamoxifen (4-OHT), or the ER subtype selective ligands, **(D)** liquiritigenin (Liq) or **(E)** methyl-piperidino-pyrazole (MPP), or the Cyclopia extracts, **(F)** SM6Met, **(G)** cup of tea (CoT) or **(H)** P104 for another 24 h. The ERα and ERβ protein levels were determined using Western blot. The western blots shown as inserts are representative of three independent experiments. For quantification, the intensity of the ERα and ERβ bands were determined with MyImage Analysis software, after which the obtained values were normalized to total protein content and expressed as a percentage (AVG ± SD) of DMSO, which was set at 100%.

**FIGURE 3:**
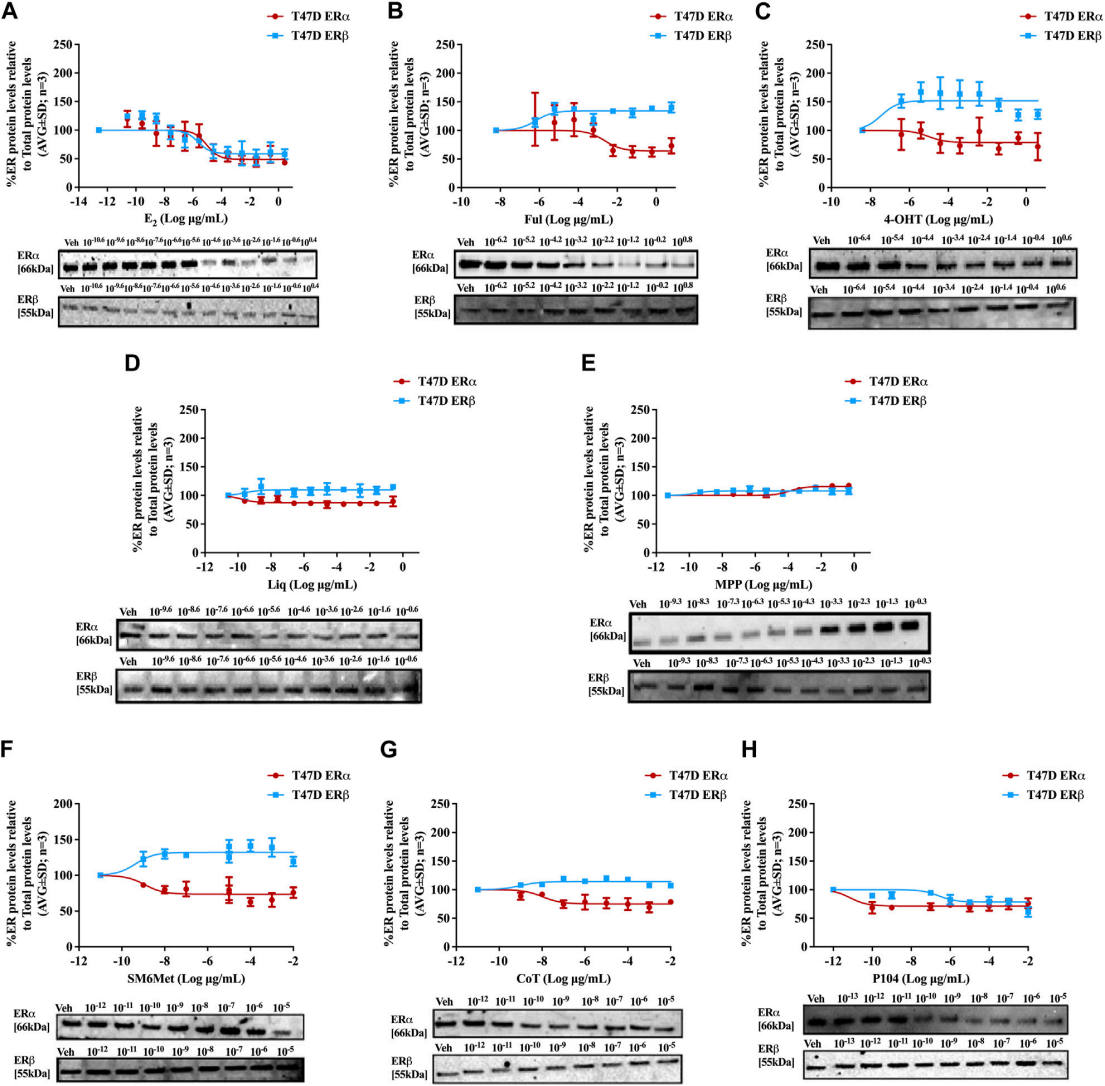
Effect of test panel on ERα and ERβ protein levels in T47D cells. T47D cells were steroid starved for 24 h and then treated with either vehicle (DMSO) or increasing concentrations of **(A)** E_2_, the SOCs, **(B)** fulvestrant (Ful) or **(C)** hydroxytamoxifen (4-OHT), or the ER subtype selective ligands, **(D)** liquiritigenin (Liq) or **(E)** methyl-piperidino-pyrazole (MPP), or the *Cyclopia* extracts, **(F)** SM6Met, **(G)** cup of tea (CoT) or **(H)** P104 for another 24 h. The ERα and ERβ protein levels were determined using Western blot. The western blots shown as inserts are representative of three independent experiments. For quantification, the intensity of the ERα and ERβ bands were determined with MyImage Analysis software, after which the obtained values were normalized to total protein content and expressed as a percentage (AVG ± SD) of DMSO, which was set at 100%.

Western blots were used to determine the efficacy and potency (Table 2) of the test panel in modulating the protein levels of ERα and ERβ in MCF7 (Figure 2) and T47D (Figure 3) cells. ERα protein levels were downregulated by all the *Cyclopia* extracts in a dose-dependent manner in the MCF7 and T47D cell lines. Specifically, for SM6Met the efficacy of the downregulation of ERα protein levels was 68.6% in MCF7 and 73.7% in T47D cells, for CoT it was 82.7% in MCF7 and 75.0% in T47D cells and for P104 it was 55.4% in MCF7 and 71.3% in T47D cells. Statistical comparison indicates that the efficacy of downregulation of ERα protein levels by the *Cyclopia* extracts was not significantly (*p* > 0.05) different (Table 2).

**TABLE 2:** Efficacy and potency of the test compounds and *Cyclopia* extracts in modulating ERα and ERβ protein levels in MCF7 and T47D cells.

Test panel	MCF7						T47D				
	ERα			ERβ		ERα:ERβ ratio^3^	ERα		ERβ		ERα:ERβ ratio
	Efficacy^1^	Potency^2^	Efficacy		Potency		Efficacy	Potency	Efficacy	Potency	
E_2_	49.3 ± 7.5^a^	−8.9 ± 0.8^a^	53.3 ± 5.6^a^		−9.3 ± 0.6^a^	0.93	48.7 ± 10.3^a^	−5.0 ± 0.8^a^^,^^b^	58.7 ± 9.0^a^	−5.6 ± 0.8^a^^,^^b^	0.83
Ful	46.8 ± 6.8^a^	−6.4 ± 0.6^a^^,^^b^	127.1 ± 9.6^b^***		−1.6 ± 0.9^b^****	0.37	64.1 ± 20.9^a^	−2.7 ± 1.8^b^	134.1 ± 4.8^b^***	−6.2 ± 0.6^a^	0.48
4-OHT	72.7 ± 10.4^a^	−4.1 ± 1.3^a^^,^^b^***	142.9 ± 8.2^b^***		−6.0 ± 0.8^a^^,^^b^**	0.51	78.8 ± 12.2^a^	−5.0 ± 2.3^a^^,^^b^	151.8 ± 10.8^b^***	−7.5 ± 2.1^a^	0.52
Liq	65.9 ± 10.9^a^	−1.9 ± 0.8^b^****	361.0 ± 36.3^d^***		−0.6 ± 0.2^b^****	0.18	87.5 ± 2.5^b^^,^^c^	−9.8 ± 1.0^a^^,^^c^**	109.7 ± 4.1^b^***	−9.5 ± 2.0^a^^,^^c^*	0.8
MPP	120.9 ± 3.0^b^***	−8.0 ± 0.7^a^	122.0 ± 2.3^b^***		−7.8 ± 0.5^a^	0.99	115.7 ± 3.4^b^**	−3.9 ± 0.7^a^^,^^b^	108.0 ± 2.1^b^***	−9.6 ± 1.5^a^^,^^c^*	1.07
SM6Met	68.6 ± 0.5^a^	−5.5 ± 1.5^a^^,^^b^*	145.4 ± 18.3^b^***		−8.8 ± 1.7^a^^,^^c^	0.47	73.7 ± 5.8^a^^,^^c^	−8.9 ± 1.0^a^^,^^c^*	132.2 ± 5.4^b^***	−9.4 ± 0.9^a^^,^^c^*	0.56
CoT	83.9 ± 11.9^a^**	−5.6 ± 2.0^a^^,^^b^*	124.5 ± 7.4^b^***		−10.0 ± 1.4^a^^,^^c^	0.66	75.0 ± 5.3^a^^,^^c^	−8.0 ± 0.8^a^^,^^c^	114.0 ± 2.4^b^***	−9.0 ± 0.7^a^ ^,c^*	0.66
P104	55.4 ± 19.2^a^	−3.3 ± 0.8^a^^,^^b^***	90.6 ± 4.0^a^^,^^c^*		−6.9 ± 1.5^a^	0.61	71.3 ± 3.4^a^^,^^c^	−11.1 ± 1.3^c^****	78.3 ± 6.4^a^^,^^c^**	−6.6 ± 1.1^a^	0.97

^1^ Efficacy expressed as AVG % ER, remaining ± SD., Values obtained from Figures 2, 3.

^2^ Potency expressed as AVG IC50 in Log μg/mL ± SD., Values obtained from Figures 2, 3. Log M values converted to Log μg/mL.

^3^ ERα:ERβ, ratio is the efficacy of ERα/efficacy of ERβ, where vehicle values are set as 100%. If = 1 it implies that the efficacy is equal *via* the ER, subtypes. If > 1 it implies that the efficacy *via* ERα > ERβ, and if < 1 it implies that the efficacy *via* ERβ > ERα.

Statistical analysis comparing the efficacy and potency between all the test compounds or extracts and both cell lines was done using One-way ANOVA with Turkey’s Multiple Comparison Test. Overall, significant difference (*p* < 0.05) between the efficacy or potency values of a test compound or extract is denoted by a different letter.

In addition, the significant difference between the effect of the test panel on the efficacy or potency within a specific ER subtype and cell line is compared to the effect of E_2_ by using Dunnett’s multiple comparisons test as post-test and indicated by *p < 0.05, **p < 0.01, ***p < 0.001.

ERβ protein levels were upregulated by the two *C. subternata* Vogel extracts, SM6Met and CoT, and downregulated by the *C. genistoides* extract, P104. Specifically, ERβ protein levels were upregulated by SM6Met to 145.4% in MCF7 and 132.2% in T47D cells, and to 124.5% in MCF7 and 114.0% in T47D cells by CoT. The upregulation of ERβ protein levels by CoT in T47D cells was lower, albeit not significantly lower than in MCF7 cells, however, it was significantly (*p* < 0.05) lower than the upregulation of ERβ protein levels by SM6Met (Table 2). In contrast, ERβ protein levels were downregulated in both cell lines by P104% to 90.56% in MCF7 and 73.8% in T47D cells, which was not statistically (*p* > 0.05) different.

Both SM6Met and CoT downregulated ERα protein levels while simultaneously increasing ERβ protein levels in both cell lines, resulting in a decreased ERα:ERβ ratio (Table 2). However, the effect of SM6Met on the ERα:ERβ ratio was greater as it was more effective at both downregulating ERα protein levels and upregulating ERβ protein levels than CoT. Although, P104 downregulated both ERα and ERβ protein levels in both cell lines, its effect on the ERα protein levels in MCF7 cells was substantially more than on the ERβ protein levels resulting in ERα:ERβ ratio reduced to about that of CoT. However, in T47D cells, the efficacies for the downregulation of the ER subtype proteins were similar and thus, P104 did not have a major influence on the ERα:ERβ ratio in T47D cells.

The potency (Table 2) of SM6Met in decreasing ERα protein levels was lower in MCF7 (3.1 × 10^−9^ mg/mL) than in T47D (1.2 × 10^−12^ mg/mL) cells as was the potency of SM6Met to increase ERβ proteins levels (1.7 × 10^−12^ mg/mL in MCF7 compared to 4.4 × 10^−13^ mg/mL in T47D cells). Similarly, the potency of CoT in decreasing ERα protein levels was lower in MCF7 (2.5 × 10^−9^ mg/mL) than in T47D (1.1 × 10^−11^ mg/mL) cells. However, in contrast, the potency of CoT in upregulating ERβ protein levels in MCF7 (1.0 × 10^−13^ mg/mL) was slightly higher than in T47D (9.9 × 10^−13^ mg/mL) cells. Additionally, the potency of P104 in decreasing ERα protein levels was significantly (*p* < 0.05) lower in MCF7 (4.6 × 10^−7^ mg/mL) than in T47D (7.1 × 10^−15^ mg/mL) cells, while the potency of P104 in downregulating ERβ protein levels in MCF7 (1.3 × 10^−10^ mg/mL) and in T47D (4.7 × 10^−10^ mg/mL) cells were similar. The potencies of the *Cyclopia* extracts in modulating either ERα or ERβ protein levels were not statistically different across the two cell lines, except for the potency of P104 in downregulating ERα protein levels in T47D cells.

Comparison of the effects elicited by the *Cyclopia* extracts with those elicited by the ER subtype specific ligands (Figures 2, 3) suggests that ERα antagonism is unlikely to be the mechanism whereby the *Cyclopia* extracts exert their SERD activity against ERα as the ERα antagonist, MPP, did not downregulate ERα. ERβ agonist activity seems a more likely mechanism for the SERD activity against ERα as the ERβ agonist, liquiritigenin, did downregulate ERα.

In comparing the relative effects of the full test panel in modulating the ERα:ERβ ratio (Table 2), we can distinguish three groups. Those that did not really affect the ratio (ERα:ERβ ratio around 1), which includes E_2_ and MPP in both cell lines, and liquiritigenin and P104 only in T47D cells. Those that had a marked effect on the ratio (ERα:ERβ ratio around 0.5), which includes the SOCs and the *Cyclopia* extracts, except for P104 in T47D cells, and those that had a major effect on the ratio (ERα:ERβ ratio below 0.2) such as liquiritigenin only in MCF7 cells. Overall comparison of the effects of the *Cyclopia* extracts with that of the SOCs indicates that the *C. subternata* Vogel extract, SM6Met, was slightly less effective than fulvestrant, but as effective as 4-OHT, in reducing the ERα:ERβ ratio, however with a markedly higher potency in increasing ERβ protein levels, while CoT was slightly less effective than both the SOCs and P104 was the least effective *Cyclopia* extract at reducing the ERα:ERβ ratio.

### 3.2 Exploration of the molecular mechanism whereby *Cyclopia* extracts modulate ERα and ERβ protein levels

Estrogenic ligands regulate the expression and stability of ERα and ERβ in BC through diverse molecular mechanisms depending on the conformation change elicited in the ER subtypes (Pink and Jordan, 1996; Khissiin and Leclercq, 1999; Wijayaratne and McDonnell, 2001). These molecular mechanisms involve the transcriptional, translational as well as the post-translational stages (Kondakova et al., 2020), through a process that may be described as a “push” versus “pull” mechanism. The “push” is controlled by transcriptional and translational processes, while the “pull” is controlled by post-translational processes that result in the degradation of the receptor protein, mediated primarily by the ubiquitin-proteasome pathway (UPS) (Kondakova et al., 2020). To explore the molecular mechanisms underlying the modulation of ERα and ERβ protein turnover by the *Cyclopia* extracts in MCF7 and T47D BC cells, translation was inhibited using a protein synthesis inhibitor, cycloheximide (CHX) (Baliga et al., 1969; Perry et al., 1995), while degradation of the ER *via* the UPS was inhibited using a proteasome inhibitor, MG132 (Khissiin and Leclercq, 1999; Fan et al., 2004).

#### 3.2.1 Effect of inhibition of protein synthesis on modulation of ER subtype protein levels by *Cyclopia* extracts

**FIGURE 4:**
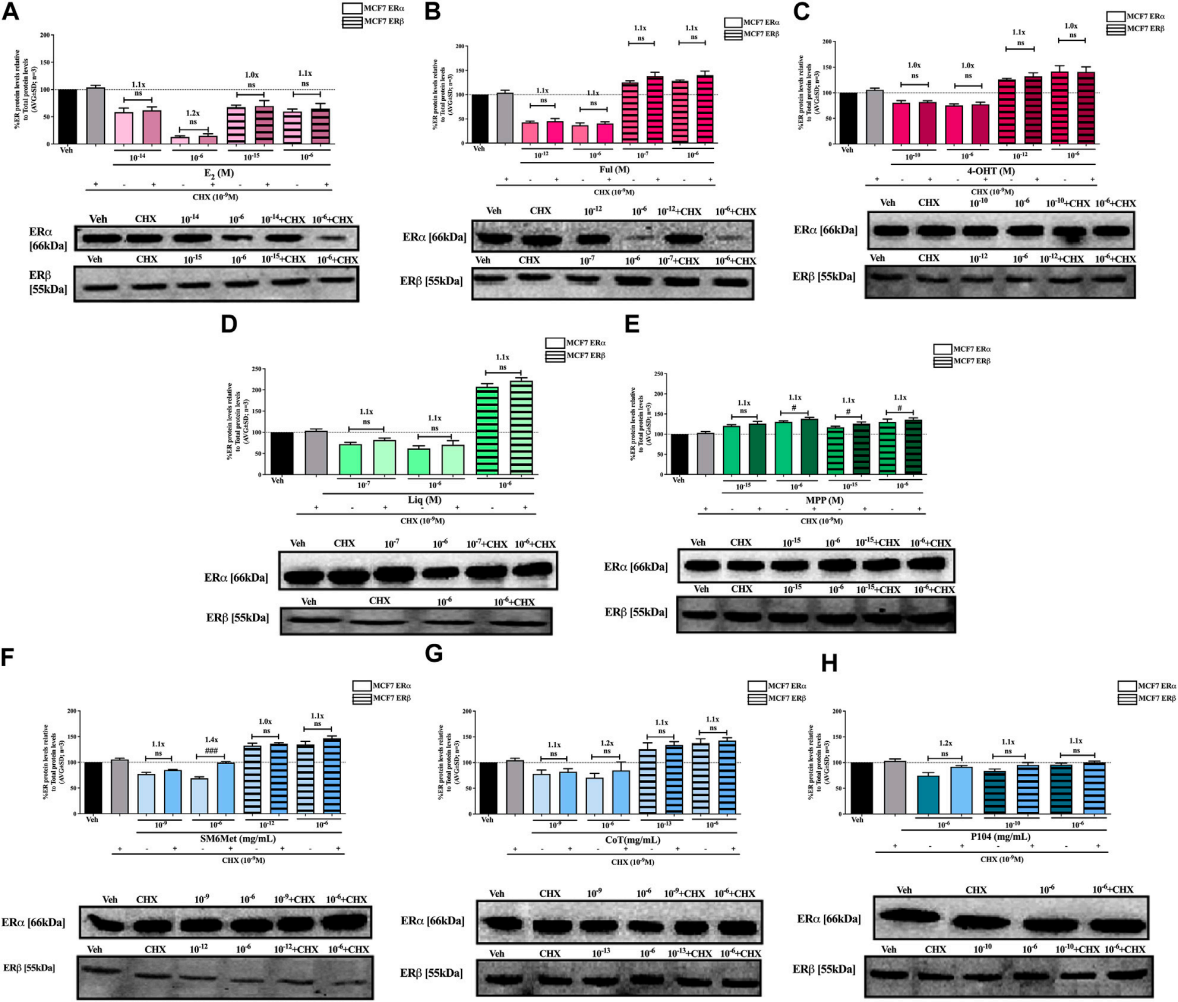
Effect of CHX, a protein synthesis inhibitor, on the modulation of ERα and ERβ protein levels in MCF7 cells. MCF7 cells were steroid starved for 24 h and then treated with either vehicle (DMSO) or LogIC_50_ (from Figure 2; Table 2 with µg/ml converted to M) and saturating (1 μM) concentrations of **(A)** E_2_ or the SOCs, **(B)** fulvestrant (Ful) or **(C)** 4-OH-tamoxifen (4-OHT), or the ER subtype selective ligands, **(D)** liquiritigenin (Liq) or **(E)** methyl-piperidino-pyrazole (MPP), or LogIC_50_ (from Figure 2 in µg/ml) and saturating (10^−6 ^μg/ml) concentrations of the *Cyclopia* extracts, **(F)** SM6Met, **(G)** cup of tea (CoT) or **(H)** P104 in the presence or absence of 1 nM CHX for another 24 h, after which the effect of ± CHX on ERα and ERβ protein levels were determined using Western blot. The western blots shown as insert are representatives of three independent experiments. For quantification, the intensity of the ERα and ERβ bands were determined with MyImage Analysis software, after which the obtained values were normalized to total protein content and expressed as a percentage (AVG ± SD) of DMSO, which was set at 100%. Fold-change is indicated above the bars. Statistical analysis was done using a two-tailed *t*-test to establish significant differences due to addition of CHX (^#^ *p* < 0.05, ^##^ *p* < 0.01 and ^###^ *p* < 0.001).

**FIGURE 5:**
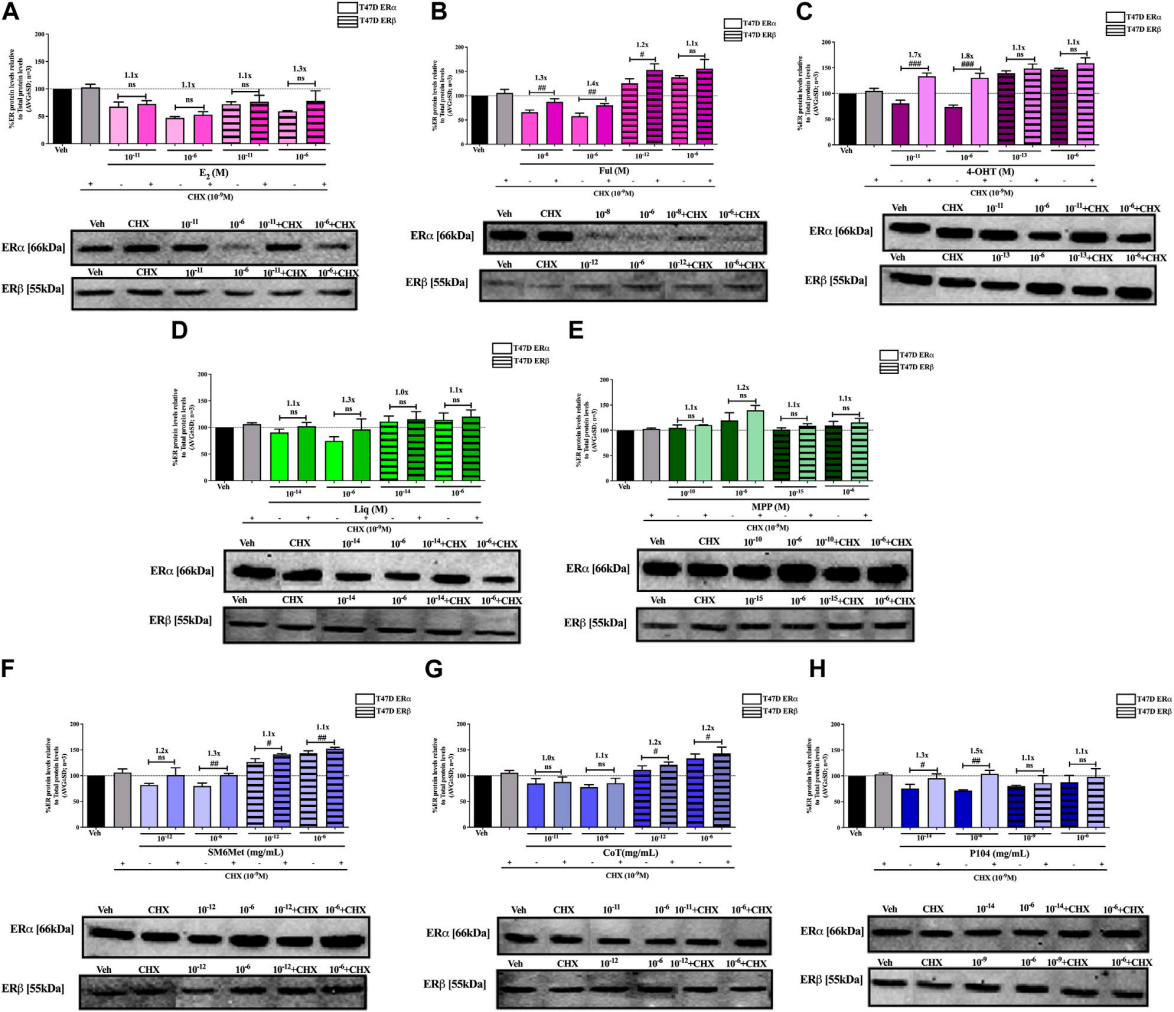
Effect of CHX, a protein synthesis inhibitor, on the modulation of ERα and ERβ protein levels in T47D cells. T47D cells were steroid starved for 24 h and then treated with either vehicle (DMSO) or LogIC_50_ (from Figure 3; Table 2 with µg/ml converted to M) and saturating (1 μM) concentrations of **(A)** E_2_ or the SOCs, **(B)** fulvestrant (Ful) or **(C)** 4-OH-tamoxifen (4-OHT), or the ER subtype selective ligands, **(D)** liquiritigenin (Liq) or **(E)** methyl-piperidino-pyrazole (MPP), or LogIC_50_ (from Figure 3 in µg/ml) and saturating (10^−6 ^μg/ml) concentrations of the *Cyclopia* extracts, **(F)** SM6Met, **(G)** cup of tea (CoT) or **(H)** P104 in the presence or absence of 1 nM CHX for another 24 h, after which the effect of ± CHX on ERα and ERβ protein levels were determined using Western blot. The western blots shown as insert are representatives of three independent experiments. For quantification, the intensity of the ERα and ERβ bands were determined with MyImage Analysis software, after which the obtained values were normalized to total protein content and expressed as a percentage (AVG ± SD) of DMSO, which was set at 100%. Fold-change is indicated above the bars. Statistical analysis was done using a two-tailed *t*-test to establish significant differences due to addition of CHX (^#^ *p* < 0.05, ^##^ *p* < 0.01 and ^###^ *p* < 0.001).

SM6Met and CoT downregulate ERα and upregulate ERβ protein levels in MCF7 and T47D cells, while P104 downregulates the protein levels of both ER subtypes (Figures 4, 5). Addition of the translational inhibitor, CHX, reversed the downregulation of ERα by SM6Met to basal levels in both MCF7 (Figure 4F) and T47D (Figure 5F) cells, however, significantly (*p* < 0.01) so only at the higher concentration of SM6Met where the increase in protein levels was between 1.3 and 1.4-fold. Similarly, CHX also reversed the downregulation of ERα by P104 to basal levels in both cell lines (Figures 4H, 5H), however, significance (*p* < 0.05) was only observed in the T47D cells where the increase in protein levels was between 1.3 and 1.5-fold. In contrast, the addition of CHX had little effect on the downregulation of ERα by CoT (Figures 4G, 5G) and only increased levels by 1.1-fold. Generally, the addition of CHX did not have a significant effect on the modulation of ERβ protein levels, except in the case of SM6Met (Figure 5F) and CoT (Figure 5G) in T47D cells where the stabilizing effect of CHX on ERβ protein levels, though small (1.1 to 1.2-fold), is significant (*p* < 0.05).

The addition of the translational inhibitor, CHX, caused no significant difference in the modulation of ERα and ERβ protein levels by liquiritigenin (Figures 4D, 5D) in both cell lines, and in the modulation by MPP (Figure 5E) in T47D cells. However, in MCF7 cells, there was a slight (1.1-fold), yet significant (*p* < 0.05), increase in the protein levels of ERα and ERβ upon the addition of CHX to MPP compared to MPP alone (Figure 4E).

Similarly, the addition of the translational inhibitor, CHX, had no significant effect on the downregulation of either ERα or ERβ protein levels by E2 in either cell line (Figures 4A, 5A). Likewise, the effect of fulvestrant (Figure 4B) and 4-OHT (Figure 4C) on ERα and ERβ protein levels in MCF7 cells was not significantly altered by the addition of CHX. However, translational inhibition through the addition of CHX significantly (*p* < 0.05) increased the protein levels of ERα and ERβ modulated by fulvestrant in T47D cells (Figure 5B) by 1.3 to 1.4-fold and 1.2-fold, respectively. Similarly, the modulation of ERα, but not ERβ, protein levels by 4-OHT in T47D cells (Figure 5C) was significantly (*p* < 0.0001) reversed (1.7 to 1.8-fold) by the addition of CHX.

#### 3.2.2 Effect of inhibition of proteasomal inhibition on modulation of ER subtype protein levels by *Cyclopia* extracts

**FIGURE 6:**
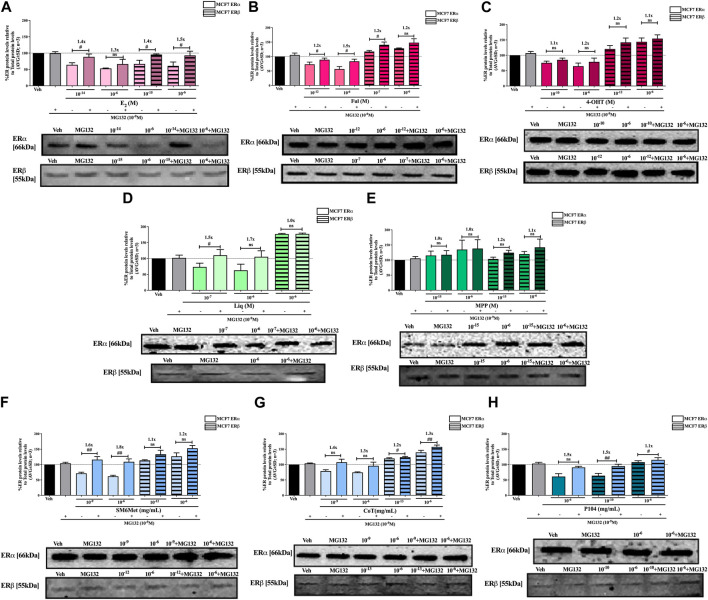
Effect of MG132, a UPS inhibitor, on the modulation of ERα and ERβ protein levels in MCF7 cells. MCF7 cells were steroid starved for 24 h and then treated with either vehicle (DMSO) or LogIC_50_ (from Figure 2; Table 2 with µg/ml converted to M) and saturating (1 μM) concentrations of **(A)** E_2_ or the SOCs, **(B)** fulvestrant (Ful) or **(C)** 4-OH-tamoxifen (4-OHT), or the ER subtype selective ligands, **(D)** liquiritigenin (Liq) or **(E)** methyl-piperidino-pyrazole (MPP), or LogIC_50_ (from Figure 2 in µg/ml) and saturating (10^−6 ^μg/ml) concentrations of the *Cyclopia* extracts, **(F)** SM6Met, **(G)** cup of tea (CoT) or **(H)** P104 in the presence or absence of 1 nM MG132 for another 24 h, after which the effect of ± MG132 on ERα and ERβ protein levels were determined using Western blot. The western blots shown as insert are representatives of three independent experiments. For quantification, the intensity of the ERα and ERβ bands were determined with MyImage Analysis software, after which the obtained values were normalized to total protein content and expressed as a percentage (AVG ± SD) of DMSO, which was set at 100%. Fold-change is indicated above the bars. Statistical analysis was done using a two-tailed *t*-test to establish significant differences due to addition of MG132 (^#^ *p* < 0.05, ^##^ *p* < 0.01 and ^###^ *p* < 0.001).

**FIGURE 7:**
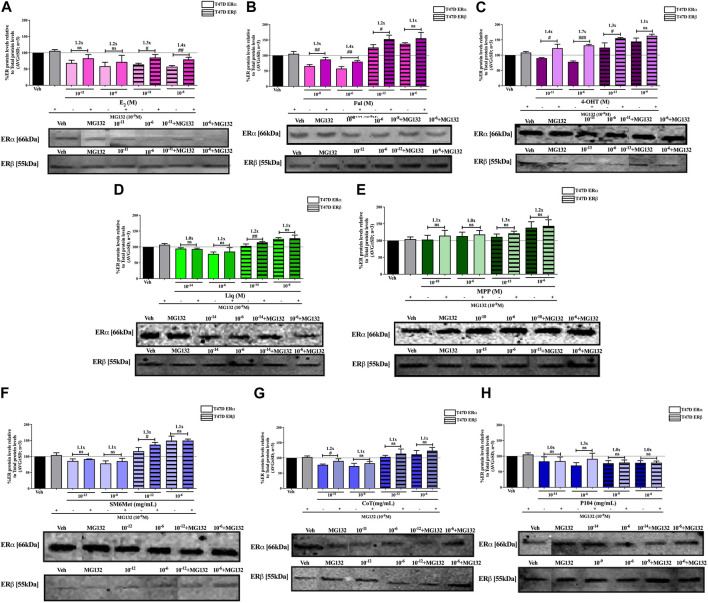
Effect of MG132, a UPS inhibitor, on the modulation of ERα and ERβ protein levels in T47D cells. T47D cells were steroid starved for 24 h and then treated with either vehicle (DMSO) or LogIC_50_ (from Figure 3; Table 2 with µg/ml converted to M) and saturating (1 μM) concentrations of **(A)** E_2_ or the SOCs, **(B)** fulvestrant (Ful) or **(C)** 4-OH-tamoxifen (4-OHT), or the ER subtype selective ligands, **(D)** liquiritigenin (Liq) or **(E)** methyl-piperidino-pyrazole (MPP), or LogIC_50_ (from Figure 3 in µg/ml) and saturating (10^−6 ^μg/ml) concentrations of the *Cyclopia* extracts, **(F)** SM6Met, **(G)** cup of tea (CoT) or **(H)** P104 in the presence or absence of 1 nM MG132 for another 24 h, after which the effect of ± MG132 on ERα and ERβ protein levels were determined using Western blot. The western blots shown as insert are representatives of three independent experiments. For quantification, the intensity of the ERα and ERβ bands were determined with MyImage Analysis software, after which the obtained values were normalized to total protein content and expressed as a percentage (AVG ± SD) of DMSO, which was set at 100%. Fold-change is indicated above the bars. Statistical analysis was done using a two-tailed *t*-test to establish significant differences due to addition of MG132 (^#^ *p* < 0.05, ^##^ *p* < 0.01 and ^###^ *p* < 0.001).

Inhibition of proteasomal degradation with MG132 generally counteracts the downregulatory effect of the *Cyclopia* extracts, SM6Met, CoT and P104, on ERα protein levels while enhancing the stabilization of ERβ protein levels by SM6Met and CoT (Figures 6, 7). Specifically, proteasomal inhibition counteracts the effects of the *Cyclopia* extracts, SM6Met (Figure 6F), CoT (Figure 6G) and P104 (Figure 6H) in downregulating ERα protein levels in MCF7 cells. Although not always significantly higher, the magnitude of the change due to the addition of MG132 was substantial (1.3 to 1.8- fold). In the T47D cells, the effects of MG132 on the downregulation of ERα protein levels by the *Cyclopia* extracts (Figures 7F–H) was substantially lower (1.1 to 1.3-fold) and mostly not significant. Stabilization of ERβ protein levels by SM6Met (Figures 6F, 7F) and CoT (Figures 6G, 7G) in both MCF7 and T47D cells was enhanced (1.1 to 1.3-fold), although not always significantly, by the addition of MG132. The effect of P104 on ERβ protein levels was significantly (*p* < 0.05) reversed (1.1 to 1.5-fold) by proteasomal inhibition in MCF7 cells (Figure 6H), while in T47D cells (Figure 7H) no effect was observed by adding MG132.

Generally, proteasomal inhibition with MG132 did not significantly counteract the effects of the ER subtype selective ligands on the ERα and ERβ protein levels (Figures 6D, E, 7D, E), apart from liquiritigenin-induced downregulation of ERα protein levels in MCF7 cells (Figure 6D) and increased stabilization of ERβ protein levels by liquiritigenin (Figure 7D) in T47D cells. Although these effects were statistically significant, the magnitude of the fold-change due to MG132 was only substantial for ERα protein levels in MCF7 cells (1.5 to 1.7-fold) but not for ERβ protein levels in T47D cells (1.1 to 1.2-fold).

Proteasomal inhibition with MG132 largely counteracts the effects of E2 (Figures 6A, 7A) in downregulating both ERα and ERβ in MCF7 and T47D cells. Although not always significantly higher, the fold change in ER levels due to MG132 addition are of a higher magnitude in MCF7 cells (1.3 to 1.5-fold) than in T47D cells (1.2 to 1.4-fold). Downregulation of ERα protein levels by the SOCs, fulvestrant (Figures 6B, 7B) and 4-OHT (Figures 6C, 7C) was counteracted to a statistically significant degree (*p* < 0.05) by the addition of MG132, except in the case of 4-OHT in MCF7 cells. Interestingly, the highest fold-change in ER levels due to the addition of MG132 (1.4 to 1.7-fold) was observed with 4-OHT in T47D cells, where addition of MG132 stabilized ERα protein levels to above that of basal levels. Upregulation of ERβ protein levels by the SOCs, fulvestrant (Figures 6B, 7B) and 4-OHT (Figures 6C, 7C), was slightly enhanced (between 1.1 and 1.3-fold) by the addition of MG132, although not always to a statistically significant degree (*p* < 0.05).

## 4 Discussion

The SOCs for ER^+^ BC include SERDs such as fulvestrant that target and reduce the expression of ERα (Lu and Liu, 2020; Hernando et al., 2021). Specifically, fulvestrant, the only SERD currently used clinically, targets and degrades ERα protein through proteasomal degradation and is often used to combat tamoxifen and AI resistance (Mottamal et al., 2021). However, due to the poor pharmacokinetics associated with fulvestrant administration, which necessitates intramuscular injection, there are limitations on its bioavailability, which results in incomplete ERα repression by fulvestrant (Croxtall and McKeage, 2011; Nathan et al., 2017). Regardless, the positive attributes of fulvestrant, by degrading ERα protein, proffer insights for the development of novel oral SERDs with improved bioavailability to overcome endocrine therapy resistance in BC with improved efficacy and potency (Lu and Liu, 2020; Hernando et al., 2021). Furthermore, not only the absolute levels of ERα but rather the levels of ERα relative to that of ERβ, the ERα:ERβ ratio, has been shown to play an important role in the BC prognosis (Evers et al., 2014; Acconcia et al., 2017). ERα facilitates cell proliferation while ERβ enables cell apoptosis and counteracts the proliferative activity of ERα (Huang et al., 2015), and thus, an increased ERα:ERβ ratio is often associated with BC (Zhao et al., 2015; Acconcia et al., 2017). Therefore, the main objective in designing a novel SERD includes an oral pharmacokinetic profile superior to that of fulvestrant and a higher efficacy and potency of ERα degradation (Lu and Liu, 2020; Shagufta et al., 2020). Furthermore, if these novel SERDs were to selectively target ERα, but not ERβ, and thereby reduce the ERα:ERβ ratio that would be an added advantage (O’Boyle et al., 2018).

From our results, it is clear that the *C. subternata* Vogel extracts, SM6Met and CoT, but not the *C. genistoides* extract, P104, display the most desirable attributes for BC prevention and treatment in downregulating ERα while upregulating ERβ and thereby reducing the ERα:ERβ ratio in both BC cell lines. Comparison of the effects on the ERα:ERβ ratio elicited by the *C. subternata* Vogel extracts, SM6Met and CoT, with those elicited by the SOCs, fulvestrant and 4-OHT, suggests that SM6Met is slightly less effective than the SERD, fulvestrant, but as effective as the SERM, 4-OHT, while CoT is less effective than both the SOCs. However, the potencies of the *C. subternata* Vogel extracts, SM6Met and CoT, are generally markedly higher than that of fulvestrant. We have previously shown that the *C. subternata* Vogel extracts, SM6Met and CoT, are absorbed when administered orally and elicit a biological effect *in vivo*, specifically by significantly reducing uterine weight and significantly delaying vaginal opening relative to solvent in the immature rat uterotrophic assay (Visser et al., 2013). Furthermore, SM6Met has demonstrated efficacy in reducing tumor mass and volume and increasing tumor free survival in a *N*-Methyl-*N*-nitrosourea (MNU)-induced rat mammary gland carcinogenesis model (Visser et al., 2016) and in suppressing tumor growth in an orthotopic model of LA7 cell-induced mammary tumors (Oyenihi et al., 2018). Thus the proven oral bioavailability of the *C. subternata* Vogel extracts coupled to the generally higher potency and comparable efficacy *in vitro* SERD activity suggest that these extracts are worthy of further investigation.

The downregulation of ERα protein levels by E2 and fulvestrant in both MCF7 and T47D cell lines agrees with previous findings (Power and Thompson, 2003; Yeh et al., 2013; Garner et al., 2015; Joseph et al., 2016; Liu et al., 2016), while the downregulation of ERα protein levels by 4-OHT in the current study contradicts some previous findings (Power and Thompson, 2003; Garner et al., 2015), but is supported by others (Koibuchi et al., 2000; Joseph et al., 2016) in an estrogen-depleted environment as also used in the current study. Specifically, Garner et al. (2015) showed that at 48 h, ERα protein levels were completely (100%) downregulated by 1 nM E2 and by 100 nM fulvestrant, while treatment with 1 μM of 4-OHT had no effect on ERα protein levels in MCF7 cells. Likewise, Yeh et al. (2013) demonstrated that after 6 h, ERα protein levels in MCF7 cells were downregulated to 35% by 100 nM of E2 and 100 nM of fulvestrant. Also, Joseph et al. (2016) performed a dose-response assay and showed that ERα protein levels were downregulated by 1 μM of fulvestrant with an efficacy of 6.4%, while 1 μM 4-OHT displayed an efficacy of 51.9% at 4 h in MCF7 cells. Furthermore, Power and Thompson (2003), demonstrated that at 24 h, ERα protein levels were downregulated by 1 nM of E2 in MCF7 cells, while no effect was seen in T47D cells. Also in the same study, 1 μM of 4-OHT was shown to upregulate ERα protein levels in both cell lines. Liu et al. (2016) showed that ERα protein levels were downgraded by more than 50% in response to fulvestrant within the concentration range of 0.03–1 μM in T47D cells. Even though our results of the downregulation of ERα protein levels by E2 and fulvestrant, in MCF7 cells agree with the findings above, comparison of the extent of the downregulation of ERα protein levels (efficacy) is difficult due to the different time points used. The discrepancies in our results showing downregulation of ERα protein levels by 4-OHT and E2, with the no effect of 1 nM E2 treatment on ERα levels demonstrated by Power and Thompson (Power and Thompson, 2003) in T47D cells, and the findings of no effect on- and the upregulation of ERα protein levels by 4-OHT for both cell lines as shown by Garner et al. (2015) and Power and Thompson (2003), respectively, may be due to the variations in the genotypes of the cell lines used by the different laboratories, the difference in the concentrations of the test compounds and experimental procedures such as different time points used for test compound treatment, as well as different culture conditions and passage number and used by the diverse laboratories (Jones et al., 2000; Bahia et al., 2002; Wenger et al., 2004; Kleensang et al., 2016). Reports on the potencies of E2, fulvestrant and 4-OHT to modulate ER subtypes are rare as few researchers attempt dose-response curves, however, Joseph et al. (2016) demonstrated that ERα protein levels were downregulated by fulvestrant with a potency of 0.39 nM, while 4-OHT showed a potency of 0.14 nM in MCF7 cells, which differs slightly from our results showing a potency for fulvestrant of 6.94 × 10^−13^ M and 2.04 × 10^−10^ M for 4-OHT.

The downregulation of ERβ protein levels by E2 in both MCF7 and T47D cell lines agrees with most previous findings (Peekhaus et al., 2004; Mishra et al., 2016), however, contradicts the findings of Power and Thompson (2003). Specifically, Mishra et al. (2016) showed that ERβ protein levels were downregulated by 1 nM E2 in MCF7 cells, while Peekhaus et al. (2004) demonstrated that ERβ protein levels were downregulated by 10 nM E2 in MCF7 cells transfected with an ERβ expression vector. In contrast, Power and Thompson (2003) showed that ERβ protein levels were significantly upregulated by 1 nM E2 in both MCF7 and T47D cells. Furthermore, the upregulation of ERβ protein levels by fulvestrant in both MCF7 and T47D cell lines agrees with Mishra et al. (2016) and Peekhaus et al. (2004). Specifically, Mishra et al. (2016) showed that ERβ protein levels were upregulated by 1 μM fulvestrant in MCF7 cells, while Peekhaus et al. (2004) demonstrated that ERβ protein levels were upregulated by 10 nM fulvestrant in MCF7 cells transfected with an ERβ expression vector. The upregulation of ERβ protein levels by 4-OHT in both MCF7 and T47D cell lines agrees with Peekhaus et al. (2004), however, contradicts the findings of Power and Thompson (2003) in T47D, but not MCF7 cells. Specifically, Peekhaus et al. (2004) demonstrated that ERβ protein levels were upregulated by 10 nM tamoxifen in MCF7 cells transfected with an ERβ expression vector. Although Power and Thompson (2003) also demonstrated that 24 h treatment of 1 μM 4-OHT significantly upregulated ERβ protein levels in MCF7 cells, they did, however, demonstrate significant downregulation in T47D cells.

To recapitulate, E2, fulvestrant and 4-OHT all downregulated ERα protein levels in a concentration-dependent manner in both cell lines, however, the extent of downregulation by 4-OHT was considerably less. In contrast, although E2 downregulated ERβ protein levels, fulvestrant and 4-OHT both significantly elevated ERβ protein levels in both cell lines. Thus, the ERα:ERβ ratio was not greatly affected by E2, however, fulvestrant and 4-OHT greatly reduced the ERα:ERβ ratio confirming their beneficial effects in ER^+^ BC (Leclercq et al., 2006; Sotoca Covaleda et al., 2008; Pons et al., 2014; Acconcia et al., 2017). Of note, the potency of fulvestrant in upregulating ERβ protein levels was significantly lower in MCF7 than in T47D cells, while the potency of downregulation of ERα protein levels was significantly higher in MCF7 than in T47D cells, which may be because of the high ERα:ERβ ratio in MCF7 and low ERα:ERβ ratio in T47D cells (Pons et al., 2014).

To the best of our knowledge, this is the first report of the dose-response modulation of ERα and ERβ protein levels by the ER subtype selective ligands, liquiritigenin and MPP, in BC cell lines. Liquiritigenin repressed ERα protein levels while concurrently increasing ERβ protein levels in both cell lines resulting in a decreased ERα:ERβ ratio. In contrast, MPP upregulated ERα and ERβ protein levels to the same extent in both cell lines and thus did not influence the ERα:ERβ ratio. Although liquiritigenin has been shown to bind to both ERα and ERβ with the same affinity, liquiritigenin specifically activates ERβ transcriptional activity and not that of ERα (Mersereau et al., 2008; Powell and Xu, 2008). Furthermore, the isomeric precursor of liquiritigenin, isoliquiritigenin (Ramalingam et al., 2018) and an extract from licorice root, which also consists of liquiritigenin, had been shown to downregulate ERα protein levels in MCF7 cells (Maggiolini et al., 2002; Hu et al., 2009), while liquiritigenin itself, as found in the current study, significantly downregulated ERα and upregulated ERβ levels in a BT-474 breast cancer cell-derived tumor xenograft model (Liang et al., 2022). The *Cyclopia* extracts all demonstrate ERα antagonism and ERβ agonism (Visser et al., 2013), however, a comparison of the effects of the ER subtype selective ligands, MPP (ERα antagonist) and liquiritigenin (ERβ agonist), suggests that the ERβ agonist rather than ERα antagonist activity of the *Cyclopia* extracts is responsible for the modulation of ER subtype levels observed.

Regarding the *Cyclopia* extracts previous work by Visser (2013) showed that 9.8 μg/mL of all three *Cyclopia* extracts downregulates ERα protein levels in MCF7 cells with efficacies of 89.8%, 86.0%, and 70.1% for SM6Met, CoT and P104, respectively. Visser did not do dose-response curves and thus potencies cannot be compared but as the concentration used by Visser corresponds to the highest concentration used during the current study, efficacies may be compared. Thus, results indicate that the efficacy for the downregulation of ERα protein levels in MCF7 cells by SM6Met at 68.6% is higher in the current study than the 89.8% shown by Visser, as is the 55.4% downregulation by P104 in the current study compared to the 70.1% shown by Visser. However, in contrast, the extent of downregulation of ERα protein levels in MCF7 cells by CoT in the current study (82.7%) is similar to the 86.0% shown by Visser (2013). Likewise, Visser (2013) showed that 9.8 μg/mL of all three *Cyclopia* extracts upregulates ERβ protein levels in MCF7 cells. The efficacy of the upregulation of ERβ protein levels in MCF7 cells by SM6Met is slightly higher at 145.4% in the current study than the 130.8% shown by Visser, as is the efficacy of CoT at 124.5% in the current study compared to the 110.9% shown by Visser. In contrast to that seen by Visser (2013), ERβ protein levels were downregulated by P104 in the current study.

Comparison of the attributes of the *Cyclopia* extracts revealed in the current study with that of other botanicals or plant extracts suggest some similarities. For example, the citrus plant-derived flavanone naringenin had been shown to have little effect on ERα (Acconcia et al., 2017) up to 1 µM but to decrease ERα protein levels at 200 µM (Xu et al., 2018), while increasing ERβ protein levels in MCF7 cells (Xu et al., 2018). Additionally, genistein, the major isoflavonoid found in soybeans, had little effect on ERα protein levels in MCF7 and T47D cells, while strongly increasing ERβ protein levels in T47D, but not MCF7 cells (Pons et al., 2014). Acetyltanshinone IIA (ATA), chemically modified from tanshinone IIA (TIIA), a major compound that was isolated from a medicinal plant, *Salvia miltiorrhiza,* specifically reduces the protein levels of ERα, but not ERβ, in MCF7 cells (Yu et al., 2014). Furthermore, triptolide, a diterpenoid isolated from the plant *Tripterygium wilfordii* Hook F also decreased ERα protein levels in MCF7 cells (Li et al., 2015), as did artemisinin, an antimalarial sesquiterpene lactone phytochemical isolated from the sweet wormwood plant, *Artemisia annua*, with the latter also shown to have no effect on ERβ protein levels in MCF7 cells (Sundar et al., 2008).

In addition, assessment of the polyphenolic compounds quantified in the *C. subternata* Vogel extracts, SM6Met and CoT, and the *C. genistoides* extract, P104, (Table 1) may provide clues to their selective ER subtype downregulation. For instance, the xanthones, mangiferin and isomangiferin, and hespiridin that are present in both *C. subternata* and *C. genistoides*, were suggested to possess anti-cancer activities (Wang et al., 2018; Hsu et al., 2021; Yap et al., 2021). Specifically, hesperidin promotes MCF7 cell proliferation in the dose range of 12.5–100 μM (Liu et al., 2008) while displaying anti-proliferative activities above 100 μM (Hsu et al., 2021), downregulates ERα mRNA levels in MCF7 and T47D cells (Khamis et al., 2018), and increases ERβ protein levels in the hypothalamus of ovariectomized mice (Han et al., 2018). Therefore, hesperidin, which is the main (2.049 g/100 g dry extract) polyphenol quantified in SM6Met, and which is present at 2.2-fold higher levels than in CoT (0.935 g/100 g dry extract), may explain the fact that the efficacy of SM6Met in downregulating ERα protein and upregulating ERβ protein levels is generally greater than that of CoT. Hesperidin was not quantified in P104.

Isomangiferin is the major polyphenol (Table 1) quantified in the *C. genistoides* extract, P104 (5.094/100 g dry extract), and is 7.9 to 12.1-fold higher than the levels in the *C. subternata* Vogel extracts, SM6Met and CoT, respectively, and although no work has been done on its effect on ERα or ERβ protein levels it has been shown to inhibit MCF7 cell proliferation and to suppress tumor growth in a mouse breast cancer mouse xenograft model using MDA-MB-231 cells (Wang et al., 2018). It would thus be interesting to evaluate the effects of isomangiferin on the ER subtype protein levels to ascertain if it is responsible for the downregulation of both ER subtypes by P104 as shown in the current study.

Mangiferin (Table 1), which is the major polyphenol (Table 1) quantified in the *C. subternata* Vogel extract, CoT (1 g/100 g dry extract), but is 1.9 to 3.6-fold lower than the levels in the *C. subternata* Vogel extract, SM6Met and the *C. genistoides* extract, P104, respectively, have been shown to activate transcription *via* ERα but not *via* ERβ (Wilkinson et al., 2015), to inhibit proliferation of MCF7 cells (Li et al., 2013; Lv et al., 2013; Cuccioloni et al., 2016; Min Yap et al., 2021) and to increase ERβ, but not ERα, mRNA expression in bone marrow macrophage cells (Sekiguchi et al., 2017). It is thus difficult to speculate what the effect of mangiferin would be on the ER subtype protein levels and this would have to be investigated in future.

Furthermore, luteolin and protocatechuic acid downregulate DHT-induced ERα protein expression and upregulate DHT-suppressed ERβ protein expression in a human prostatic epithelial cell line, BPH-1 (Tao et al., 2019), while luteolin reduces ERα protein expression in MCF7 cells (Wang et al., 2012), selectively transactivates *via* ERβ but not *via* ERα in SK-N-BE neuroblastoma cells (Innocenti et al., 2007), but not in HEK293 cells transfected with ER subtypes (Mortimer et al., 2015), inhibits E2-induced ERα transactivation in a yeast assay (Pinto et al., 2008), binds preferentially to ERβ (Verhoog et al., 2007b) and displays partial agonist activity in stimulating MCF7 cell proliferation (Resende et al., 2013) but inhibits E2-induced proliferation in MCF7 cells (Verhoog et al., 2007b). Luteolin is, however, present at very low concentrations in all *Cyclopia* extracts and is thus unlikely to alone be responsible for the effects of the *Cyclopia* extracts. In fact, we have previously shown that activity-guided fractionation does not retain all the desirable estrogenic attributes of the original SM6Met in one fraction (Mortimer et al., 2015) and thus it maybe the combinatorial effect of all or several of the compounds in the extracts that contribute to the selective modulation of the ER subtypes. Although some isolated pure phytoestrogen compounds are active against BC, it has been postulated that the range of their activity is less compared to that of crude extracts as the multifactorial reactions and synergy between phytoestrogenic compounds are only present in crude extracts (Gilbert and Alves, 2005; Rasoanaivo et al., 2011). Thus, phytoestrogenic extracts rather than isolated phytoestrogens may increase the likelihood of combining the attributes, such as the ability to downregulate ERα, upregulate ERβ and preferentially decrease the ERα:ERβ ratio, thought to be desirable for BC treatment and prevention.

Taken together, our findings show that the molecular mechanism involved in the regulation of ERα and ERβ protein levels may be organized into several types. Those primarily regulated through proteasomal degradation such as E2 and liquiritigenin and those such as MPP primarily regulated through transcriptional and translational mechanisms. Other types involve a mixture of mechanisms, either equally or preferentially leaning towards one of the mechanisms. Specifically, 4-OHT and the *Cyclopia* extracts, CoT and P104, appear to equally favor proteasomal, and transcriptional and translational mechanisms, while fulvestrant and SM6Met generally favor proteasomal degradation.

The regulation of ERα protein levels *via* proteasomal degradation by E2 and fulvestrant in MCF7 cells agrees with the findings of Zhao et al. (2015), and Wijayaratne and McDonnell (2001), while regulation of ERβ protein levels *via* proteasomal degradation by E2 agrees with the findings of Zhao et al. (2015). Furthermore, Khissiin and Leclercq (Khissiin and Leclercq, 1999) showed that the downregulation of ERα protein levels by E2 in MCF7 cells was *via* both protein synthesis and proteasomal degradation. Additionally, although not in BC cells, Alarid et al. (1999) using CHX and MG132, and the transcription inhibitor, 5,6-DRB demonstrated that ERα protein levels downregulation by E2 was through proteasomal degradation and not *via* protein synthesis nor transcription in lactotrope cells, PR1. Our report is the first on the molecular mechanism of regulation of ER subtypes by the *Cyclopia* extracts and to our knowledge also by the ER subtype selective ligands, liquiritigenin and MPP.

Despite many studies investigating the selective degradation of the ER for BC treatment (Lu and Liu, 2020; Shagufta et al., 2020; Wang Z. et al., 2021; Kumar et al., 2021; Mottamal et al., 2021), these have mostly focused on ERα with little mention of ERβ. Our study in elaborating on the molecular characteristics and mechanism of action of the *Cyclopi*a extracts has in contrast explicitly evaluated selectively in terms of ER subtype levels. In conclusion, the current study indicates that the *C. subternata* Vogel extracts, SM6Met and CoT, rather than the *C. genistoides* extract, P104, display favorable attributes by degrading ERα while stabilizing ERβ. Coupled to the proven oral bioavailability of the *C. subternata* Vogel extracts (Visser et al., 2013; Visser et al., 2016; Oyenihi et al., 2018) the current study suggests that the *C. subternata* Vogel extracts may be of therapeutic benefit for BC prevention and treatment and provide the underpinning for the development of an ER-targeted phytopharmaceutical product from *Cyclopia*.

## Data Availability

The original contributions presented in the study are included in the article/Supplementary Material, further inquiries can be directed to the corresponding author.

## References

[B1] Acconcia F. Fiocchetti M. Marino M. (2017). Xenoestrogen regulation of ERα/ERβ balance in hormone-associated cancers. Mol. Cell. Endocrinol. 457, 3–12. 10.1016/j.mce.2016.10.033 27816767

[B2] Alarid E. T. Bakopoulos N. Solodin N. (1999). Proteasome-mediated proteolysis of estrogen receptor: A novel component in autologous down-regulation. Mol. Endocrinol. 13 (9), 1522–1534. 10.1210/mend.13.9.0337 10478843

[B3] Bahia H. Ashman J. N. Cawkwell L. Lind M. Monson J. R. Drew P. J. (2002). Karyotypic variation between independently cultured strains of the cell line MCF-7 identified by multicolour fluorescence *in situ* hybridization. Int. J. Oncol. 20 (3), 489–494. 10.3892/ijo.20.3.489 11836559

[B4] Baliga B. S. Pronczuk A. W. Munro H. N. (1969). Mechanism of cycloheximide inhibition of protein synthesis in a cell-free system prepared from rat liver. J. Biol. Chem. 244 (16), 4480–4489. 10.1016/s0021-9258(18)94343-7 5806588

[B5] Beelders T. de Beer D. Stander M. A. Joubert E. (2014). Comprehensive phenolic profiling of Cyclopia genistoides (L.) Vent. by LC-DAD-MS and -MS/MS reveals novel xanthone and benzophenone constituents. Molecules 19 (8), 11760–11790. 10.3390/molecules190811760 25105916 PMC6271833

[B6] Chellan N. Joubert E. Strijdom H. Roux C. Louw J. Muller C. J. F. (2014). Aqueous extract of unfermented honeybush (cyclopia maculata) attenuates stz-induced diabetes and β-cell cytotoxicity. Planta Med. 80 (8–9), 622–629. 10.1055/s-0034-1368457 24853761

[B7] Chintamunnee V. Mahomoodally M. F. (2012). Herbal medicine commonly used against non-communicable diseases in the tropical island of Mauritius. J. Herb. Med. 2 (4), 113–125. 10.1016/j.hermed.2012.06.001

[B8] Chopra A. Willmore W. G. Biggar K. K. (2019). Protein quantification and visualization via ultraviolet-dependent labeling with 2,2,2-trichloroethanol. Sci. Rep. 9 (1), 13923. 10.1038/s41598-019-50385-9 31558752 PMC6763483

[B9] Clarke R. Tyson J. J. Dixon J. M. (2015). Endocrine resistance in breast cancer-An overview and update. Mol. Cell. Endocrinol. 418 (0 3), 220–234. Pt 3. 10.1016/j.mce.2015.09.035 26455641 PMC4684757

[B10] Costa B. Amorim I. Gärtner F. Vale N. (2020). Understanding breast cancer: From conventional therapies to repurposed drugs. Eur. J. Pharm. Sci. 151, 105401. 10.1016/j.ejps.2020.105401 32504806

[B11] Croxtall J. D. McKeage K. (2011). Fulvestrant: A review of its use in the management of hormone receptor-positive metastatic breast cancer in postmenopausal women. Fulvestrant. *Drugs* 71 (3), 363–380. 10.2165/11204810-000000000-00000 21319872

[B12] Cuccioloni M. Bonfili L. Mozzicafreddo M. Cecarini V. Scuri S. Cocchioni M. (2016). Mangiferin blocks proliferation and induces apoptosis of breast cancer cells: Via suppression of the mevalonate pathway and by proteasome inhibition. Food Funct. 7, 4299–4309. 10.1039/c6fo01037g 27722367

[B13] Dalglish S. L. Straubinger S. Kavle J. A. Gibson L. Mbombeshayi E. Anzolo J. (2019). Who are the real community health workers in Tshopo Province, Democratic Republic of the Congo? BMJ Glob. Health 4 (4), e001529–9. 10.1136/bmjgh-2019-001529 PMC6615876 31354973

[B14] Datta J. Willingham N. Manouchehri J. M. Schnell P. Sheth M. David J. J. (2022). Activity of estrogen receptor β agonists in therapy-resistant estrogen receptor-positive breast cancer. Front. Oncol. 12, 857590. 10.3389/fonc.2022.857590 35574319 PMC9097292

[B15] DeSantis C. E. Bray F. Ferlay J. Lortet-Tieulent J. Anderson B. O. Jemal A. (2015). International variation in female breast cancer incidence and mortality rates. Cancer Epidemiol. Biomarkers Prev. 24 (10), 1495–1506. 10.1158/1055-9965.EPI-15-0535 26359465

[B16] DeSantis C. E. Ma J. Gaudet M. M. Newman L. A. Miller K. D. Goding Sauer A. (2019). Breast cancer statistics, 2019. CA Cancer J. Clin. 69 (6), 438–451. 10.3322/caac.21583 31577379

[B17] Downton T. Zhou F. Segara D. Jeselsohn R. Lim E. (2022). Oral selective estrogen receptor degraders (SERDs) in breast cancer: Advances, challenges, and current status. Drug Des. Devel Ther. 16, 2933–2948. 10.2147/DDDT.S380925 PMC9447452 36081610

[B18] Ekor M. (2014). The growing use of herbal medicines: Issues relating to adverse reactions and challenges in monitoring safety. Front. Neurol. 4, 1–10. 10.3389/fphar.2013.00177 PMC3887317 24454289

[B19] Evers N. M. van den Berg J. H. J. Wang S. Melchers D. Houtman R. de Haan L. H. J. (2014). Cell proliferation and modulation of interaction of estrogen receptors with coregulators induced by ERα and ERβ agonists. J. Steroid Biochem. Mol. Biol. 143, 376–385. 10.1016/j.jsbmb.2014.06.002 24923734

[B20] Fan M. Nakshatri H. Nephew K. P. (2004). Inhibiting proteasomal proteolysis sustains estrogen receptor-α activation. Mol. Endocrinol. 18 (11), 2603–2615. 10.1210/me.2004-0164 15284335

[B21] Farkas S. Szabó A. Hegyi A. E. Török B. Fazekas C. L. Ernszt D. (2022). Estradiol and estrogen-like alternative therapies in use: The importance of the selective and non-classical actions. Biomedicines 10 (4), 861. 10.3390/biomedicines10040861 35453610 PMC9029610

[B22] Franzoi M. A. Agostinetto E. Perachino M. del Mastro L. de Azambuja E. Vaz-Luis I. (2021). Evidence-based approaches for the management of side-effects of adjuvant endocrine therapy in patients with breast cancer. Lancet Oncol. 22 (7), e303–e313. 10.1016/S1470-2045(20)30666-5 33891888

[B23] Garner F. Shomali M. Paquin D. Lyttle C. R. Hattersley G. (2015). RAD1901: A novel, orally bioavailable selective estrogen receptor degrader that demonstrates antitumor activity in breast cancer xenograft models. Anticancer Drugs 26 (9), 948–956. 10.1097/CAD.0000000000000271 26164151 PMC4560273

[B24] Gilbert B. Alves L. (2005). Synergy in plant medicines. Curr. Med. Chem. 10 (1), 13–20. 10.2174/0929867033368583 12570718

[B25] Girgert R. Emons G. Gründker C. (2019). Estrogen signaling in erα-negative breast cancer: ERβ and GPER. Front. Endocrinol. (Lausanne) 10 (JAN), 781. 10.3389/fendo.2018.00781 30687231 PMC6333678

[B26] Gonzalez T. L. Rae J. M. Colacino J. A. (2019). Implication of environmental estrogens on breast cancer treatment and progression. Toxicology 421, 41–48. 10.1016/j.tox.2019.03.014 30940549 PMC6561091

[B27] Gurib-Fakim A. (2006). Medicinal plants: Traditions of yesterday and drugs of tomorrow. Mol. Asp. Med. 27 (1), 1–93. 10.1016/j.mam.2005.07.008 16105678

[B28] Gustafsson J. A. Warner M. (2000). Estrogen receptor beta in the breast: Role in estrogen responsiveness and development of breast cancer. J. Steroid Biochem. Mol. Biol. 74 (5), 245–248. Available at: http://www.ncbi.nlm.nih.gov/pubmed/11162931 (Accessed December 13, 2018). 10.1016/s0960-0760(00)00130-8 11162931

[B29] Han N. R. Nam S. Y. Hong S. Kim H. Y. Moon P. D. Kim H. J. (2018). Improvement effects of a mixed extract of flowers of Pueraria thomsonii Benth. and peels of Citrus unshiu Markovich on postmenopausal symptoms of ovariectomized mice. Biomed. Pharmacother. 103, 524–530. 10.1016/j.biopha.2018.04.070 29677538

[B30] Hartman J. Ström A. Gustafsson J. Å. (2009). Estrogen receptor beta in breast cancer-Diagnostic and therapeutic implications. Steroids 74 (8), 635–641. 10.1016/j.steroids.2009.02.005 19463683

[B31] Hernando C. Ortega-Morillo B. Tapia M. Moragón S. Martínez M. T. Eroles P. (2021). Oral selective estrogen receptor degraders (Serds) as a novel breast cancer therapy: Present and future from a clinical perspective. Int. J. Mol. Sci. 22 (15), 7812. 10.3390/ijms22157812 34360578 PMC8345926

[B32] Hirao-Suzuki M. (2021). Estrogen receptor β as a possible double-edged sword molecule in breast cancer: A mechanism of alteration of its role by exposure to endocrine-disrupting chemicals. Biol. Pharm. Bull. 44 (11), 1594–1597. 10.1248/bpb.b21-00468 34719637

[B33] Hsu P. H. Chen W. H. Juanlu C. Hsieh S. C. Lin S. C. Mai R. T. (2021). Hesperidin and chlorogenic acid synergistically inhibit the growth of breast cancer cells via estrogen receptor/mitochondrial pathway. Life 11 (9), 950. 10.3390/life11090950 34575098 PMC8467139

[B34] Hu C. Liu H. Du J. Mo B. Qi H. Wang X. (2009). Estrogenic activities of extracts of Chinese licorice (Glycyrrhiza uralensis) root in MCF-7 breast cancer cells. J. Steroid Biochem. Mol. Biol. 113 (3–5), 209–216. 10.1016/j.jsbmb.2008.12.019 19167497

[B35] Huang B. Warner M. Gustafsson J. Å. (2015). Estrogen receptors in breast carcinogenesis and endocrine therapy. Mol. Cell. Endocrinol. 418, 240–244. 10.1016/j.mce.2014.11.015 25433206

[B36] Human C. Danton O. de Beer D. Maruyama T. Alexander L. Malherbe C. (2021). Identification of a novel di-C-glycosyl dihydrochalcone and the thermal stability of polyphenols in model ready-to-drink beverage solutions with Cyclopia subternata extract as functional ingredient. Food Chem. 351, 129273. 10.1016/j.foodchem.2021.129273 33662907

[B37] Innocenti G. Vegeto E. Dall’Acqua S. Ciana P. Giorgetti M. Agradi E. (2007). *In vitro* estrogenic activity of *Achillea millefolium* L. Phytomedicine 14 (2–3), 147–152. 10.1016/j.phymed.2006.05.005 16860978

[B38] Joubert E. de Beer D. Malherbe C. J. J. Muller M. Louw A. Gelderblom W. C. A. C. A. (2019). Formal honeybush tea industry reaches 20-year milestone – progress of product research targeting phenolic composition, quality and bioactivity. South Afr. J. Bot. 127, 58–79. 10.1016/j.sajb.2019.08.027

[B39] Jack B. U. Malherbe C. J. Willenburg E. L. De Beer D. Huisamen B. Joubert E. (2018). Polyphenol-enriched fractions of cyclopia intermedia selectively affect lipogenesis and lipolysis in 3T3-L1 adipocytes. Planta Med. 84 (2), 100–110. 10.1055/s-0043-119463 28938495

[B40] Jones C. Payne J. Wells D. Delhanty J. D. A. Lakhani S. R. Kortenkamp A. (2000). Comparative genomic hybridization reveals extensive variation among different MCF-7 cell stocks. Cancer Genet. Cytogenet 117 (2), 153–158. 10.1016/S0165-4608(99)00158-2 10704689

[B41] Jordan V. C. (2003). Tamoxifen: A most unlikely pioneering medicine. Nat. Rev. Drug Discov. 2 (3), 205–213. 10.1038/nrd1031 12612646

[B42] Joseph J. D. Darimont B. Zhou W. Arrazate A. Young A. Ingalla E. (2016). The selective estrogen receptor downregulator GDC-0810 is efficacious in diverse models of ER+ breast cancer. Elife 5 (JULY 13), e15828. 10.7554/eLife.15828 27410477 PMC4961458

[B43] Joubert E. Gelderblom W. C. A. C. A. Louw A. de Beer D. (2008). South African herbal teas: Aspalathus linearis, Cyclopia spp. and Athrixia phylicoides-A review. J. Ethnopharmacol. 119 (3), 376–412. 10.1016/j.jep.2008.06.014 18621121

[B44] Keydar I. Chen L. Karby S. Weiss F. R. Delarea J. Radu M. (1979). Establishment and characterization of a cell line of human breast carcinoma origin. Eur. J. Cancer 15 (5), 659–670. 10.1016/0014-2964(79)90139-7 228940

[B45] Khamis A. A. A. Ali E. M. M. El-Moneim M. A. A. Abd-Alhaseeb M. M. El-Magd M. A. Salim E. I. (2018). Hesperidin, piperine and bee venom synergistically potentiate the anticancer effect of tamoxifen against breast cancer cells. Biomed. Pharmacother. 105, 1335–1343. 10.1016/j.biopha.2018.06.105 30021371

[B46] Khissiin A. Leclercq G. (1999). Implication of proteasome in estrogen receptor degradation. FEBS Lett. 448 (1), 160–166. 10.1016/S0014-5793(99)00343-9 10217432

[B47] Kleensang A. Vantangoli M. M. Odwin-Dacosta S. Andersen M. E. Boekelheide K. Bouhifd M. (2016). Genetic variability in a frozen batch of MCF-7 cells invisible in routine authentication affecting cell function. Sci. Rep. 6, 28994. 10.1038/srep28994 27456714 PMC4960662

[B48] Koibuchi Y. Iino Y. Uchida T. Andoh T. Horii Y. Nagasawa M. (2000). Regulation of estrogen receptor and epidermal growth factor receptor by tamoxifen under high and low estrogen environments in MCF-7 cells grown in athymic mice. Oncol. Rep. 7 (1), 135–140. 10.3892/or.7.1.135 10601607

[B49] Kondakova I. v. Shashova E. E. Sidenko E. A. Astakhova T. M. Zakharova L. A. Sharova N. P. (2020). Estrogen receptors and ubiquitin proteasome system: Mutual regulation. Biomolecules 10 (4), 500. 10.3390/biom10040500 32224970 PMC7226411

[B50] Kumar N. Gulati H. K. Sharma A. Heer S. Jassal A. K. Arora L. (2021). Most recent strategies targeting estrogen receptor alpha for the treatment of breast cancer. Mol. Divers 25 (1), 603–624. 10.1007/s11030-020-10133-y 32886304

[B51] Lacroix M. Leclercq G. (2004). Relevance of breast cancer cell lines as models for breast tumours: An update. Breast Cancer Res. Treat. 83 (3), 249–289. 10.1023/B:BREA.0000014042.54925.cc 14758095

[B52] Leclercq G. Lacroix M. Laios I. Laurent G. (2006). Estrogen receptor alpha: Impact of ligands on intracellular shuttling and turnover rate in breast cancer cells. Curr. Cancer Drug Targets 6 (1), 39–64. 10.2174/156800906775471716 16475975

[B53] Leung Y. K. Lee M. T. Lam H. M. Tarapore P. Ho S. M. (2012). Estrogen receptor-beta and breast cancer: Translating biology into clinical practice. Steroids 77 (7), 727–737. 10.1016/j.steroids.2012.03.008 22465878 PMC3356459

[B54] Li H. Huang J. Yang B. Xiang T. Yin X. Peng W. (2013). Mangiferin exerts antitumor activity in breast cancer cells by regulating matrix metalloproteinases, epithelial to mesenchymal transition, and β-catenin signaling pathway. Toxicol. Appl. Pharmacol. 272 (1), 180–190. 10.1016/j.taap.2013.05.011 23707762

[B55] Li H. Pan G. F. Jiang Z. Z. Yang J. Sun L. X. Zhang L. Y. (2015). Triptolide inhibits human breast cancer MCF-7 cell growth via downregulation of the ERα-mediated signaling pathway. Acta Pharmacol. Sin. 36 (5), 606–613. 10.1038/aps.2014.162 25864647 PMC4422943

[B56] Liang Y. Besch-Williford C. Hyder S. M. (2022). The estrogen receptor beta agonist liquiritigenin enhances the inhibitory effects of the cholesterol biosynthesis inhibitor RO 48-8071 on hormone-dependent breast-cancer growth. Breast Cancer Res. Treat. 192 (1), 53–63. 10.1007/s10549-021-06487-y 35037188

[B57] Liu J. Zheng S. Akerstrom V. L. Yuan C. Ma Y. Zhong Q. (2016). Fulvestrant-3 boronic acid (ZB716): An orally bioavailable selective estrogen receptor downregulator (SERD). J. Med. Chem. 59 (17), 8134–8140. 10.1021/acs.jmedchem.6b00753 27529700 PMC5499704

[B58] Liu L. Xu D. M. Cheng Y. Y. (2008). Distinct effects of naringenin and hesperetin on nitric oxide production from endothelial cells. J. Agric. Food Chem. 56 (3), 824–829. 10.1021/jf0723007 18197618

[B59] Lopes C. M. Dourado A. Oliveira R. (2017). Phytotherapy and nutritional supplements on breast cancer. Biomed. Res. Int. 2017, 7207983. 10.1155/2017/7207983 28845434 PMC5563402

[B60] Louw A. Joubert E. Visser K. (2013). Phytoestrogenic potential of cyclopia extracts and polyphenols. Planta Med. 79 (7), 580–590. 10.1055/s-0032-1328463 23609108

[B61] Lu Y. Liu W. (2020). Selective estrogen receptor degraders (SERDs): A promising strategy for estrogen receptor positive endocrine-resistant breast cancer. J. Med. Chem. 63 (24), 15094–15114. 10.1021/acs.jmedchem.0c00913 33138369

[B62] Lv J. Wang Z. Zhang L. Wang H. L. Liu Y. Li C. (2013). Mangiferin induces apoptosis and cell cycle arrest in MCF-7 cells both *in vitro* and *in vivo* . J. Animal Veterinary Adv. 12 (3), 352–359. 10.3923/javaa.2013.352-359

[B63] Maggiolini M. Statti G. Vivacqua A. Gabriele S. Rago V. Loizzo M. (2002). Estrogenic and antiproliferative activities of isoliquiritigenin in MCF7 breast cancer cells. J. Steroid Biochem. Mol. Biol. 82 (4–5), 315–322. 10.1016/S0960-0760(02)00230-3 12589938

[B64] Mal R. Magner A. David J. Datta J. Vallabhaneni M. Kassem M. (2020). Estrogen receptor beta (ERβ): A ligand activated tumor suppressor. Front. Oncol. 10, 587386. 10.3389/fonc.2020.587386 33194742 PMC7645238

[B65] Mbendana D. Mamabolo K. Truter M. Kritzinger Q. Ndhlala A. R. (2019). Practices at herbal (muthi) markets in gauteng, South Africa and their impact on the health of the consumers: A case study of KwaMai-mai and marabastad muthi markets. South Afr. J. Bot. 126, 30–39. 10.1016/j.sajb.2019.05.004

[B66] Mersereau J. E. Levy N. Staub R. E. Baggett S. Zogric T. Chow S. (2008). Liquiritigenin is a plant-derived highly selective estrogen receptor β agonist. Mol. Cell. Endocrinol. 283 (1–2), 49–57. 10.1016/j.mce.2007.11.020 18177995 PMC2277338

[B67] Mfenyana C. DeBeer D. Joubert E. Louw A. (2008). Selective extraction of Cyclopia for enhanced *in vitro* phytoestrogenicity and benchmarking against commercial phytoestrogen extracts. J. Steroid Biochem. Mol. Biol. 112 (1–3), 74–86. 10.1016/j.jsbmb.2008.08.005 18793725

[B68] Min Yap K. Sekar M. Jing Seow L. Hua Gan S. Reddy Bonam S. Najihah Izzati Mat Rani N. (2021). Mangifera indica (mango): A promising medicinal plant for breast cancer therapy and understanding its potential mechanisms of action. 10.2147/BCTT.S316667 PMC8448164 34548817

[B69] Mishra A. K. Abrahamsson A. Dabrosin C. (2016). Fulvestrant inhibits growth of triple negative breast cancer and synergizes with tamoxifen in ERα positive breast cancer by up-regulation of ERβ. Oncotarget 7 (35), 56876–56888. 10.18632/oncotarget.10871 27486755 PMC5302959

[B70] Mortimer M. Visser K. de Beer D. Joubert E. Louw A. (2015). Divide and conquer may not be the optimal approach to retain the desirable estrogenic attributes of the cyclopia nutraceutical extract, SM6Met. PLoS One 10 (7), e0132950. 10.1371/journal.pone.0132950 26208351 PMC4514865

[B71] Mottamal M. Kang B. Peng X. Wang G. (2021). From pure antagonists to pure degraders of the estrogen receptor: Evolving strategies for the same target. ACS Omega 6 (14), 9334–9343. 10.1021/acsomega.0c06362 33869913 PMC8047716

[B72] Murakami S. Miura Y. Hattori M. Matsuda H. Malherbe C. J. Muller C. J. F. (2018). Cyclopia extracts enhance Th1-Th2-and Th17-type T cell responses and induce Foxp3 + cells in murine cell culture. Planta Med. 84 (5), 311–319. 10.1055/s-0043-121270 29096404

[B73] Nadal-Serrano M. Sastre-Serra J. Pons D. G. Miró A. M. Oliver J. Roca P. (2012). The ERalpha/ERbeta ratio determines oxidative stress in breast cancer cell lines in response to 17Beta-estradiol. J. Cell. Biochem. 113 (10), 3178–3185. 10.1002/jcb.24192 22615145

[B74] Nathan M. R. Schmid P. Schmid M. R. N. P. (2017). A review of fulvestrant in breast cancer. Oncol. Ther. 5 (1), 17–29. 10.1007/s40487-017-0046-2 28680952 PMC5488136

[B75] Nilsson S. Koehler K. F. Gustafsson J. Å. (2011). Development of subtype-selective oestrogen receptor-based therapeutics. Nat. Rev. Drug Discov. 10 (10), 778–792. 10.1038/nrd3551 21921919

[B76] O’Boyle N. M. Barrett I. Greene L. M. Carr M. Fayne D. Twamley B. (2018). Lead optimization of benzoxepin-type selective estrogen receptor (ER) modulators and downregulators with subtype-specific ERα and ERβ activity. J. Med. Chem. 61 (2), 514–534. 10.1021/acs.jmedchem.6b01917 28426931

[B77] Oyenihi O. R. Krygsman A. Verhoog N. de Beer D. Saayman M. J. Mouton T. M. (2018). Chemoprevention of LA7-induced mammary tumor growth by SM6Met, a well-characterized Cyclopia extract. Front. Pharmacol. 9, 650. 10.3389/fphar.2018.00650 29973879 PMC6019492

[B78] Pal K. (2021). Retracing our path towards mother nature: A cost-effective green therapy for the ameliorative of recalcitrant triple negative breast cancer - a brief report. Acad. Lett. 1466. 10.20935/al1466

[B79] Peekhaus N. T. Chang T. Hayes E. C. Wilkinson H. A. Mitra S. W. Schaeffer J. M. (2004). Distinct effects of the antiestrogen Faslodex on the stability of estrogen receptors-α and -β in the breast cancer cell line MCF-7. J. Mol. Endocrinol. 32 (3), 987–995. 10.1677/jme.0.0320987 15171727

[B80] Pelkonen O. Xu Q. Fan T. P. (2014). Why is research on herbal medicinal products important and how can we improve its quality? J. Tradit. Complement. Med. 4 (1), 1–7. 10.4103/2225-4110.124323 24872927 PMC4032837

[B81] Perry R. R. Kang Y. Greaves B. (1995). Effects of tamoxifen on growth and apoptosis of estrogen-dependent and -independent human breast cancer cells. Ann. Surg. Oncol. 2 (3), 238–245. 10.1007/BF02307030 7641021

[B82] Pheiffer C. Dudhia Z. Louw J. Muller C. Joubert E. (2013). Cyclopia maculata (honeybush tea) stimulates lipolysis in 3T3-L1 adipocytes. Phytomedicine 20 (13), 1168–1171. 10.1016/j.phymed.2013.06.016 23880330

[B83] Pink J. J. Jordan V. C. (1996). Models of estrogen receptor regulation by estrogens and antiestrogens in breast cancer cell lines. Cancer Res. 56 (10), 2321–2330. 8625307

[B84] Pinto B. Bertoli A. Noccioli C. Garritano S. Reali D. Pistelli L. (2008). Estradiol-antagonistic activity of phenolic compounds from leguminous plants. Phytotherapy Res. 22 (3), 362–366. 10.1002/ptr.2327 18167044

[B85] Pons D. G. Nadal-Serrano M. Blanquer-Rossello M. M. Sastre-Serra J. Oliver J. Roca P. (2014). Genistein modulates proliferation and mitochondrial functionality in breast cancer cells depending on ERalpha/ERbeta ratio. J. Cell. Biochem. 115 (5), 949–958. 10.1002/jcb.24737 24375531

[B86] Powell E. Xu W. (2008). Intermolecular interactions identify ligand-selective activity of estrogen receptor alpha/beta dimers. Proc. Natl. Acad. Sci. 105 (48), 19012–19017. 10.1073/pnas.0807274105 19022902 PMC2596243

[B87] Power K. A. Thompson L. U. (2003). Ligand-induced regulation of ERalpha and ERbeta is indicative of human breast cancer cell proliferation. Breast Cancer Res. Treat. 81 (3), 209–221. 10.1023/A:1026114501364 14620916

[B88] Ramalingam M. Kim H. Lee Y. il Lee Y. il (2018). Phytochemical and pharmacological role of liquiritigenin and isoliquiritigenin from radix glycyrrhizae in human health and disease models. Front. Aging Neurosci. 10, 348. 10.3389/fnagi.2018.00348 30443212 PMC6221911

[B89] Ramani K. v. Ramani H. Alurkar S. Ajaikumar B. S. Trivedi R. G. (2017). Breast cancer: Medical treatment, side effects, and complementary therapies. New York: Momentum Press.

[B90] Rasoanaivo P. Wright C. W. Willcox M. L. Gilbert B. (2011). Whole plant extracts versus single compounds for the treatment of malaria: Synergy and positive interactions. Malar. J. 10, S4. 10.1186/1475-2875-10-S1-S4 21411015 PMC3059462

[B91] Resende F. A. de Oliveira A. P. S. de Camargo M. S. Vilegas W. Varanda E. A. (2013). Evaluation of estrogenic potential of flavonoids using a recombinant yeast strain and MCF7/BUS cell proliferation assay. PLoS One 8 (10), e74881. 10.1371/journal.pone.0074881 24098354 PMC3788058

[B92] Roza O. Lai W. C. Zupkó I. Hohmann J. Jedlinszki N. Chang F. R. (2017). Bioactivity guided isolation of phytoestrogenic compounds from Cyclopia genistoides by the pER8:GUS reporter system. South Afr. J. Bot. 110, 201–207. 10.1016/j.sajb.2016.06.001

[B93] Rozeboom B. Dey N. De P. (2019). ER+ metastatic breast cancer: Past, present, and a prescription for an apoptosis-targeted future. Am. J. Cancer Res. 9 (12), 2821–2831. Available at: www.ajcr.us/ISSN:2156-6976/ajcr0103100 . 31911865 PMC6943351

[B94] Saha T. Makar S. Swetha R. Gutti G. Singh S. K. (2019). Estrogen signaling: An emanating therapeutic target for breast cancer treatment. Eur. J. Med. Chem. 177, 116–143. 10.1016/j.ejmech.2019.05.023 31129450

[B95] Sayed R. E. Jamal L. E. Iskandarani S. E. Kort J. Abdel Salam M. Assi H. (2019). Endocrine and targeted therapy for hormone-receptor-positive, HER2-negative advanced breast cancer: Insights to sequencing treatment and overcoming resistance based on clinical trials. Front. Oncol. 9, 510. 10.3389/fonc.2019.00510 31281796 PMC6597942

[B96] Schneider-Poetsch T. Ju J. Eyler D. E. Dang Y. Bhat S. Merrick W. C. (2010). Inhibition of eukaryotic translation elongation by cycloheximide and lactimidomycin. Nat. Chem. Biol. 6 (3), 209–217. 10.1038/nchembio.304 20118940 PMC2831214

[B97] Schulze A. E. De Beer D. Mazibuko S. E. Muller C. J. F. Roux C. Willenburg E. L. (2016). Assessing similarity analysis of chromatographic fingerprints of Cyclopia subternata extracts as potential screening tool for *in vitro* glucose utilisation. Anal. Bioanal. Chem. 408 (2), 639–649. 10.1007/s00216-015-9147-7 26542834

[B98] Sekiguchi Y. Mano H. Nakatani S. Shimizu J. Kataoka A. Ogura K. (2017). Mangiferin positively regulates osteoblast differentiation and suppresses osteoclast differentiation. Mol. Med. Rep. 16 (2), 1328–1332. 10.3892/mmr.2017.6752 28627701 PMC5562065

[B99] Shagufta Ahmad I. Mathew S. Rahman S. (2020). Recent progress in selective estrogen receptor downregulators (SERDs) for the treatment of breast cancer. RSC Med. Chem. 11 (4), 438–454. 10.1039/c9md00570f 33479648 PMC7580774

[B100] Song P. Li Y. Dong Y. Liang Y. Qu H. Qi D. (2019). Estrogen receptor β inhibits breast cancer cells migration and invasion through CLDN6-mediated autophagy. J. Exp. Clin. Cancer Res. 38 (1), 354. 10.1186/s13046-019-1359-9 31412908 PMC6694553

[B101] Soto A. M. Sonnenschein C. Chung K. L. Fernandez M. F. Olea N. Olea Serrano F. (1995). The E-SCREEN assay as a tool to identify estrogens: An update on estrogenic environmental pollutants. Environ. Health Perspect. 103, 113–122. 10.1289/ehp.95103s7113 PMC1518887 8593856

[B102] Sotoca Covaleda A. M. van den Berg H. Vervoort J. van der Saag P. Ström A. Gustafsson J.-Å. (2008). Influence of cellular ERalpha/ERbeta ratio on the ERalpha-agonist induced proliferation of human T47D breast cancer cells. Toxicol. Sci. 105 (2), 303–311. 10.1093/toxsci/kfn141 18644836 PMC2527638

[B103] Sundar S. N. Marconett C. N. Doan V. B. Willoughby J. A. Firestone G. L. (2008). Artemisinin selectively decreases functional levels of estrogen receptor-alpha and ablates estrogen-induced proliferation in human breast cancer cells. Carcinogenesis 29 (12), 2252–2258. 10.1093/carcin/bgn214 18784357 PMC2639250

[B104] Szostakowska M. Trębińska-Stryjewska A. Grzybowska E. A. Fabisiewicz A. (2019). Resistance to endocrine therapy in breast cancer: Molecular mechanisms and future goals. Breast Cancer Res. Treat. 173 (3), 489–497. 10.1007/s10549-018-5023-4 30382472 PMC6394602

[B105] Tahvilian R. Shahriari S. Faramarzi A. Komasi A. (2014). Ethno-pharmaceutical formulations in Kurdish ethno-medicine. Iran. J. Pharm. Res. 13 (3), 1029–1039. 25276205 PMC4177625

[B106] Tao R. Miao L. Yu X. Orgah J. O. Barnabas O. Chang Y. (2019). Cynomorium songaricum Rupr demonstrates phytoestrogenic or phytoandrogenic like activities that attenuates benign prostatic hyperplasia via regulating steroid 5-α-reductase. J. Ethnopharmacol. 235, 65–74. 10.1016/j.jep.2019.01.038 30708032

[B107] van Dyk L. (2018). Combinatorial treatments of tamoxifen with SM6Met, a selective estrogen receptor subtype modulator (SERSM), from Cyclopia subternata are superior to current endocrine treatments in breast cancer cell models. Available at: http://hdl.handle.net/10019.1/105232 (Accessed December 12, 2022).

[B108] van Wyk A. S. Prinsloo G. (2018). Medicinal plant harvesting, sustainability and cultivation in South Africa. Biol. Conserv. 227, 335–342. 10.1016/j.biocon.2018.09.018

[B109] Verhoog N. J. D. Joubert E. Louw A. (2007a). Evaluation of the phytoestrogenic activity of Cyclopia genistoides (honeybush) methanol extracts and relevant polyphenols. J. Agric. Food Chem. 55 (11), 4371–4381. 10.1021/jf063588n 17461595

[B110] Verhoog N. J. D. Joubert E. Louw A. (2007b). Screening of four Cyclopia (honeybush) species for putative phyto-oestrogenic activity by oestrogen receptor binding assays. S Afr. J. Sci. 103 (1–2), 13–21.

[B111] Veziari Y. Kumar S. Leach M. (2021). Addressing barriers to the conduct and application of research in complementary and alternative medicine: A scoping review. BMC Complement. Med. Ther. 21 (1), 201. 10.1186/s12906-021-03371-6 34266441 PMC8281683

[B112] Visagie A. Kasonga A. Deepak V. Moosa S. Marais S. Kruger M. C. (2015). Commercial honeybush (cyclopia spp.) tea extract inhibits osteoclast formation and bone resorption in RAW264.7 murine macrophages—an *in vitro* study. Int. J. Environ. Res. Public Health 12 (11), 13779–13793. 10.3390/ijerph121113779 26516894 PMC4661614

[B113] Visser J. A. K. (2013). Phytoestrogenic extracts of cyclopia modulate molecular targets involved in the prevention and treatment of breast cancer. Available at: http://hdl.handle.net/10019.1/86718 (Accessed December 12, 2022).

[B114] Visser K. Mortimer M. Louw A. (2013). Cyclopia extracts act as ERα antagonists and ERβ agonists, *in vitro* and *in vivo* . PLoS One 8 (11), e79223. 10.1371/journal.pone.0079223 24223909 PMC3817056

[B115] Visser K. Zierau O. Macejová D. Goerl F. Muders M. Baretton G. B. G. B. (2016). The phytoestrogenic Cyclopia extract, SM6Met, increases median tumor free survival and reduces tumor mass and volume in chemically induced rat mammary gland carcinogenesis. J. Steroid Biochem. Mol. Biol. 163, 129–135. 10.1016/j.jsbmb.2016.04.019 27142456

[B116] Wang B. Shen J. Wang Z. Liu J. Ning Z. Hu M. (2018). Isomangiferin, a novel potent vascular endothelial growth factor receptor 2 kinase inhibitor, suppresses breast cancer growth, metastasis and angiogenesis. J. Breast Cancer 21 (1), 11–20. 10.4048/jbc.2018.21.1.11 29628979 PMC5880961

[B117] Wang K. Chen Q. Shao Y. Yin S. Liu C. Liu Y. (2021). Anticancer activities of TCM and their active components against tumor metastasis. Biomed. Pharmacother. 133, 111044. 10.1016/j.biopha.2020.111044 33378952

[B118] Wang L. M. Xie K. P. Huo H. N. Shang F. Zou W. Xie M. J. (2012). Luteolin inhibits proliferation induced by IGF-1 pathway dependent ERα in human breast cancer MCF-7 cells. Asian Pac. J. Cancer Prev. 13 (4), 1431–1437. 10.7314/APJCP.2012.13.4.1431 22799344

[B119] Wang Z. Ma Z. Shen Z. (2021). Selective degradation of the estrogen receptor in the treatment of cancers. J. Steroid Biochem. Mol. Biol. 209, 105848. 10.1016/j.jsbmb.2021.105848 33610801

[B120] Wangkheirakpam S. (2018). “Traditional and folk medicine as a target for drug discovery,” in Natural products and drug discovery (Amsterdam, Netherlands: Elsevier), 29–56.

[B121] Wenger S. L. Senft J. R. Sargent L. M. Bamezai R. Bairwa N. Grant S. G. (2004). Comparison of established cell lines at different passages by karyotype and comparative genomic hybridization. Biosci. Rep. 24 (6), 631–639. 10.1007/s10540-005-2797-5 16158200

[B122] WHO Report (2019). WHO Global report on traditional and complementary medicine 2019. Geneva, Switzerland: World Health Organization.

[B123] Wijayaratne A. L. McDonnell D. P. (2001). The human estrogen receptor-α is a ubiquitinated protein whose stability is affected differentially by agonists, antagonists, and selective estrogen receptor modulators. J. Biol. Chem. 276 (38), 35684–35692. 10.1074/jbc.M101097200 11473106

[B124] Wilkinson A. S. Taing M. W. Pierson J. T. Lin C. N. Dietzgen R. G. Shaw P. N. (2015). Estrogen modulation properties of mangiferin and quercetin and the mangiferin metabolite norathyriol. Food Funct. 6 (6), 1847–1854. 10.1039/c5fo00133a 25940566

[B125] Xu Z. Huang B. Liu J. Wu X. Luo N. Wang X. (2018). Combinatorial anti-proliferative effects of tamoxifen and naringenin: The role of four estrogen receptor subtypes. Toxicology 410, 231–246. 10.1016/j.tox.2018.08.013 30153467

[B126] Yang L. N. Wu Z. L. Yang Z. J. Li S. G. Ouyang C. S. (2021). Exploring mechanism of key Chinese herbal medicine on breast cancer by data mining and network Pharmacology methods. Chin. J. Integr. Med. 27 (12), 919–926. 10.1007/s11655-020-3422-y 32572780

[B127] Yap K. M. Sekar M. Seow L. J. Gan S. H. Bonam S. R. Mat Rani N. N. I. (2021). Mangifera indica (mango): A promising medicinal plant for breast cancer therapy and understanding its potential mechanisms of action. Breast Cancer Targets Ther. 13, 471–503. 10.2147/BCTT.S316667 PMC8448164 34548817

[B128] Yeh W. L. Shioda K. Coser K. R. Rivizzigno D. McSweeney K. R. Shioda T. (2013). Fulvestrant-induced cell death and proteasomal degradation of estrogen receptor α protein in MCF-7 cells require the CSK c-src tyrosine kinase. PLoS One 8 (4), e60889. 10.1371/journal.pone.0060889 23593342 PMC3617152

[B129] Yu T. Zhou Z. Mu Y. de Lima Lopes G. Luo K. Q. (2014). A novel anti-cancer agent, acetyltanshinone IIA, inhibits oestrogen receptor positive breast cancer cell growth by down-regulating the oestrogen receptor. Cancer Lett. 346 (1), 94–103. 10.1016/j.canlet.2013.12.023 24374015

[B130] Zhao Z. Wang L. James T. Jung Y. Kim I. Tan R. (2015). Reciprocal regulation of ERα and ERβ stability and activity by diptoindonesin G. Chem. Biol. 22 (12), 1608–1621. 10.1016/j.chembiol.2015.10.011 26670079 PMC4767166

[B131] Zhou H.-B. Carlson K. E. Stossi F. Katzenellenbogen B. S. Katzenellenbogen J. A. (2009). Analogs of methyl-piperidinopyrazole (MPP): Antiestrogens with estrogen receptor alpha selective activity. Bioorg Med. Chem. Lett. 19 (1), 108–110. 10.1016/j.bmcl.2008.11.006 19014882 PMC2711511

[B132] Zink A. Traidl-Hoffmann C. (2015). Green tea in dermatology - myths and facts. JDDG J. der Deutschen Dermatologischen Gesellschaft 13 (8), 768–775. 10.1111/ddg.12737 26177066

