# Supplementary material for: Cyclopia extracts act as selective estrogen receptor subtype downregulators in estrogen receptor positive breast cancer cell lines: Comparison to standard of care breast cancer endocrine therapies and a selective estrogen receptor agonist and antagonist
Source: Front Pharmacol. 2023 Mar 13;14:1122031. doi: 10.3389/fphar.2023.1122031 (PMC10040842; doi:10.3389/fphar.2023.1122031)
Supplement: Supplementary file 1 [file DataSheet1.docx]

Supplementary Material

# Supplementary Figures and Tables

## Supplementary Figures


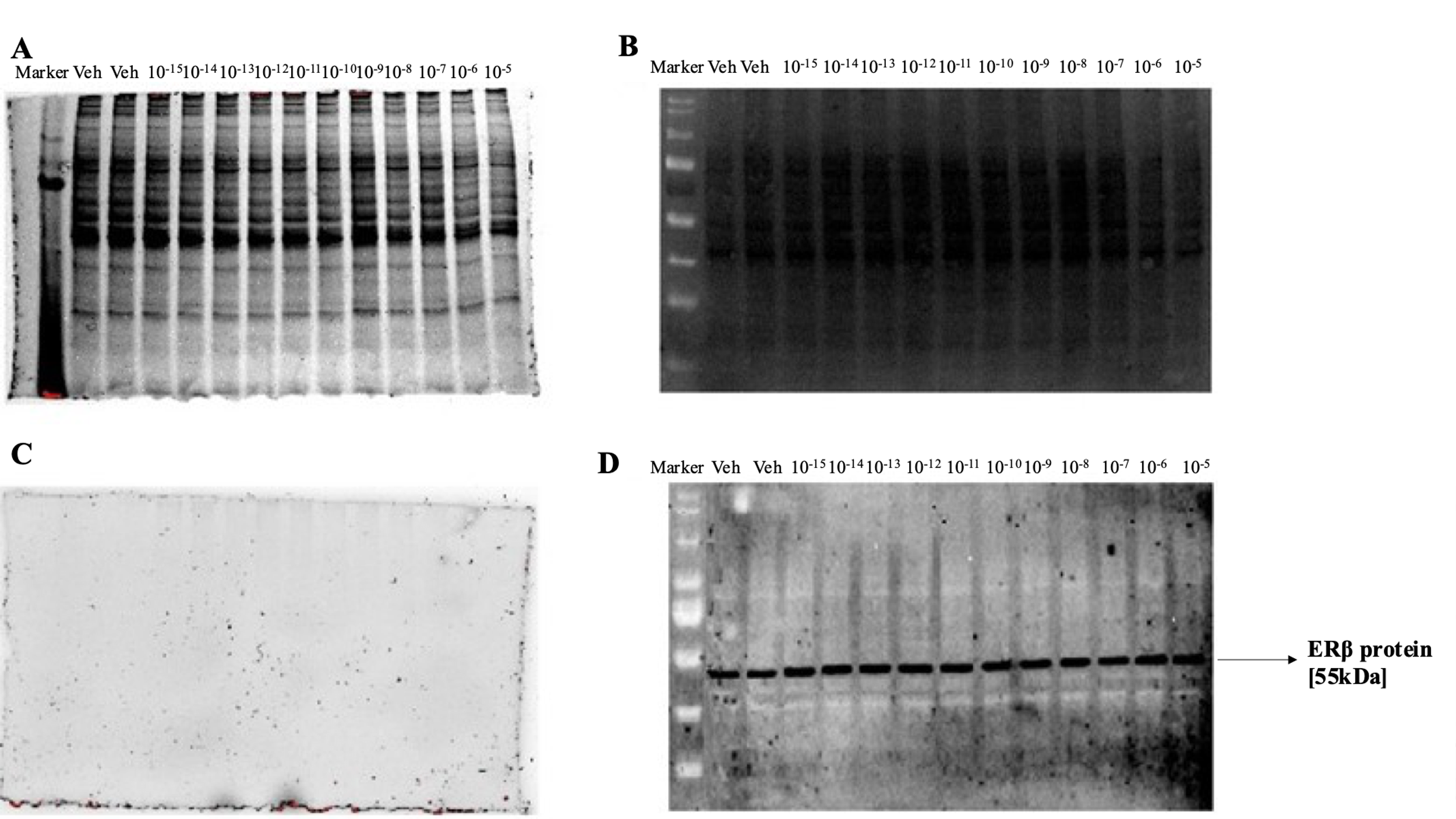


**Supplementary Figure 1.** An example of the workflow used for western blot. (**A**) contains the full SDS-PAGE gel following protein separation, (**B**) the full nitrocellulose membrane after protein transfer, (**C**) the full SDS-PAGE gel after protein transfer and (**D**) the full nitrocellulose membrane following immunoblotting, which indicates the 55kDa ERβ protein.

## Supplementary Tables

**Supplementary Table 1: Stock solutions of the test panel used.**

| **E_2_** | | | | **Ful** | | | | **4-OHT** | | | |
| --- | --- | --- | --- | --- | --- | --- | --- | --- | --- | --- | --- |
| **MCF7** | | **T47D** | | **MCF7** | | **T47D** | | **MCF7** | | **T47D** | |
| **M** | **µg/mL** | **M** | **µg/mL** | **M** | **µg/mL** | **M** | **µg/mL** | **M** | | **µg/mL** | |
| 1 x 10^-12^ | 2.75 x 10^-7^ | 1 x 10^-13^ | 2.75 x 10^-8^ | 1 x 10^-9^ | 6.07 x 10^-4^ | 1 x 10^-9^ | 6.07 x 10^-4^ | 1 x 10^-9^ | 3.89 x 10^-4^ | 1 x 10^-9^ | 3.89 x 10^-4^ |
| 1 x 10^-11^ | 2.75 x 10^-6^ | 1 x 10^-12^ | 2.75 x 10^-7^ | 1 x 10^-8^ | 6.07 x 10^-3^ | 1 x 10^-8^ | 6.07 x 10^-3^ | 1 x 10^-8^ | 3.89 x 10^-3^ | 1 x 10^-8^ | 3.89 x 10^-3^ |
| 1 x 10^-10^ | 2.75 x 10^-5^ | 1 x 10^-11^ | 2.75 x 10^-6^ | 1 x 10^-7^ | 6.07 x 10^-2^ | 1 x 10^-7^ | 6.07 x 10^-2^ | 1 x 10^-7^ | 3.89 x 10^-2^ | 1 x 10^-7^ | 3.89 x 10^-2^ |
| 1 x 10^-9^ | 2.75 x 10^-4^ | 1 x 10^-10^ | 2.75 x 10^-5^ | 1 x 10^-6^ | 6.07 x 10^-1^ | 1 x 10^-6^ | 6.07 x 10^-1^ | 1 x 10^-6^ | 3.89 x 10^-1^ | 1 x 10^-6^ | 3.89 x 10^-1^ |
| 1 x 10^-8^ | 2.75 x 10^-3^ | 1 x 10^-9^ | 2.75 x 10^-4^ | 1 x 10^-5^ | 6.07 x 10^0^ | 1 x 10^-5^ | 6.07 x 10^0^ | 1 x 10^-5^ | 3.89 x 10^0^ | 1 x 10^-5^ | 3.89 x 10^0^ |
| 1 x 10^-7^ | 2.75 x 10^-2^ | 1 x 10^-8^ | 2.75 x 10^-3^ | 1 x 10^-4^ | 6.07 x 10^+1^ | 1 x 10^-4^ | 6.07 x 10^+1^ | 1 x 10^-4^ | 3.89 x 10^+1^ | 1 x 10^-4^ | 3.89 x 10^+1^ |
| 1 x 10^-6^ | 2.75 x 10^-1^ | 1 x 10^-7^ | 2.75 x 10^-2^ | 1 x 10^-3^ | 6.07 x 10^+2^ | 1 x 10^-3^ | 6.07 x 10^+2^ | 1 x 10^-3^ | 3.89 x 10^+2^ | 1 x 10^-3^ | 3.89 x 10^+2^ |
| 1 x 10^-5^ | 2.75 x 10^0^ | 1 x 10^-6^ | 2.75 x 10^-1^ | 1 x 10^-2^ | 6.07 x 10^+3^ | 1 x 10^-2^ | 6.07 x 10^+3^ | 1 x 10^-2^ | 3.89 x 10^+3^ | 1 x 10^-2^ | 3.89 x 10^+3^ |
| 1 x 10^-4^ | 2.75 x 10^+1^ | 1 x 10^-5^ | 2.75 x 10^0^ |  | |  | |  | |  | |
| 1 x 10^-3^ | 2.75 x 10^+2^ | 1 x 10^-4^ | 2.75 x 10^+1^ |  |  |  |  |  |  |  |  |
| 1 x 10^-2^ | 2.75 x 10^+3^ | 1 x 10^-3^ | 2.75 x 10^+2^ |  |  |  |  |  |  |  |  |
|  | | 1 x 10^-2^ | 2.75 x 10^+3^ |  |  |  |  |  |  |  |  |

| **Liq** | | | | **MPP** | | | |
| --- | --- | --- | --- | --- | --- | --- | --- |
| **MCF7** | | **T47D** | | **MCF7** | | **T47D** | |
| **M** | **µg/mL** | **M** | **µg/mL** | **M** | **µg/mL** | **M** | **µg/mL** |
| 1 x 10^-12^ | 2.56 x 10^-7^ | 1 x 10^-12^ | 2.56 x 10^-7^ | 1 x 10^-11^ | 4.68 x 10^-6^ | 1 x 10^-12^ | 4.68 x 10^-7^ |
| 1 x 10^-11^ | 2.56 x 10^-6^ | 1 x 10^-11^ | 2.56 x 10^-6^ | 1 x 10^-10^ | 4.68 x 10^-5^ | 1 x 10^-11^ | 4.68 x 10^-6^ |
| 1 x 10^-10^ | 2.56 x 10^-5^ | 1 x 10^-10^ | 2.56 x 10^-5^ | 1 x 10^-9^ | 4.68 x 10^-4^ | 1 x 10^-10^ | 4.68 x 10^-5^ |
| 1 x 10^-9^ | 2.56 x 10^-4^ | 1 x 10^-9^ | 2.56 x 10^-4^ | 1 x 10^-8^ | 4.68 x 10^-3^ | 1 x 10^-9^ | 4.68 x 10^-4^ |
| 1 x 10^-8^ | 2.56 x 10^-3^ | 1 x 10^-8^ | 2.56 x 10^-3^ | 1 x 10^-7^ | 4.68 x 10^-2^ | 1 x 10^-8^ | 4.68 x 10^-3^ |
| 1 x 10^-7^ | 2.56 x 10^-2^ | 1 x 10^-7^ | 2.56 x 10^-2^ | 1 x 10^-6^ | 4.68 x 10^-1^ | 1 x 10^-7^ | 4.68 x 10^-2^ |
| 1 x 10^-6^ | 2.56 x 10^-1^ | 1 x 10^-6^ | 2.56 x 10^-1^ | 1 x 10^-5^ | 4.68 x 10^0^ | 1 x 10^-6^ | 4.68 x 10^-1^ |
| 1 x 10^-5^ | 2.56 x 10^0^ | 1 x 10^-5^ | 2.56 x 10^0^ | 1 x 10^-4^ | 4.68 x 10^+1^ | 1 x 10^-5^ | 4.68 x 10^0^ |
| 1 x 10^-4^ | 2.56 x 10^+1^ | 1 x 10^-4^ | 2.56 x 10^+1^ | 1 x 10^-3^ | 4.68 x 10^+2^ | 1 x 10^-4^ | 4.68 x 10^+1^ |
| 1 x 10^-3^ | 2.56 x 10^+2^ | 1 x 10^-3^ | 2.56 x 10^+2^ | 1 x 10^-2^ | 4.68 x 10^+3^ | 1 x 10^-3^ | 4.68 x 10^+2^ |
| 1 x 10^-2^ | 2.56 x 10^+3^ |  |  |  |  |  |  |

| **SM6Met** | | **CoT** | | **P104** | |
| --- | --- | --- | --- | --- | --- |
| **MCF7** | **T47D** | **MCF7** | **T47D** | **MCF7** | **T47D** |
| **µg/mL** | **µg/mL** | **µg/mL** | **µg/mL** | **µg/mL** | **µg/mL** |
| 1 x 10^-7^ | 1 x 10^-6^ | 1 x 10^-7^ | 1 x 10^-6^ | 1 x 10^-6^ | 1 x 10^-7^ |
| 1 x 10^-6^ | 1 x 10^-5^ | 1 x 10^-6^ | 1 x 10^-5^ | 1 x 10^-5^ | 1 x 10^-6^ |
| 1 x 10^-5^ | 1 x 10^-4^ | 1 x 10^-5^ | 1 x 10^-4^ | 1 x 10^-4^ | 1 x 10^-5^ |
| 1 x 10^-4^ | 1 x 10^-3^ | 1 x 10^-4^ | 1 x 10^-3^ | 1 x 10^-3^ | 1 x 10^-4^ |
| 1 x 10^-3^ | 1 x 10^-2^ | 1 x 10^-3^ | 1 x 10^-2^ | 1 x 10^-2^ | 1 x 10^-3^ |
| 1 x 10^-2^ | 1 x 10^-1^ | 1 x 10^-2^ | 1 x 10^-1^ | 1 x 10^-1^ | 1 x 10^-2^ |
| 1 x 10^-1^ | 1 | 1 x 10^-1^ | 1 | 1 | 1 x 10^-1^ |
| 1 | 1 x 10^+1^ | 1 | 1 x 10^+1^ | 1 x 10^+1^ | 1 |
| 1 x 10^+1^ |  | 1 x 10^+1^ |  |  | 1 x 10^+1^ |
